# Diverse immunoglobulin gene usage and convergent epitope targeting in neutralizing antibody responses to SARS-CoV-2

**DOI:** 10.1016/j.celrep.2021.109109

**Published:** 2021-04-24

**Authors:** Xiaojuan Zhou, Fengge Ma, Jun Xie, Meng Yuan, Yunqiao Li, Namir Shaabani, Fangzhu Zhao, Deli Huang, Nicholas C. Wu, Chang-Chun D. Lee, Hejun Liu, Jiali Li, Zhonghui Chen, Yazhen Hong, Wen-Hsien Liu, Nengming Xiao, Dennis R. Burton, Haijian Tu, Hang Li, Xin Chen, John R. Teijaro, Ian A. Wilson, Changchun Xiao, Zhe Huang

**Affiliations:** 1 State Key Laboratory of Cellular Stress Biology, Innovation Center for Cell Signaling Network, School of Life Sciences, Xiamen University, Xiamen, Fujian 361102, China; 2 Department of Integrative Structural and Computational Biology, The Scripps Research Institute, La Jolla, CA 92037, USA; 3 Department of Immunology and Microbiology, The Scripps Research Institute, La Jolla, CA 92037, USA; 4 Affiliated Hospital of Putian University, Putian, Fujian 351100, China; 5 Ragon Institute of Massachusetts General Hospital, Massachusetts Institute of Technology and Harvard University, Cambridge, MA 02139, USA; 6 The Skaggs Institute for Chemical Biology, The Scripps Research Institute, La Jolla, CA 92037, USA

**Keywords:** SARS-CoV-2, neutralizing antibody, diverse immunoglobulin gene usage, convergent epitope targeting

## Abstract

It is unclear whether individuals with enormous diversity in B cell receptor repertoires are consistently able to mount effective antibody responses against SARS-CoV-2. We analyzed antibody responses in a cohort of 55 convalescent patients and isolated 54 potent neutralizing monoclonal antibodies (mAbs). While most of the mAbs target the angiotensin-converting enzyme 2 (ACE2) binding surface on the receptor binding domain (RBD) of SARS-CoV-2 spike protein, mAb 47D1 binds only to one side of the receptor binding surface on the RBD. Neutralization by 47D1 is achieved independent of interfering RBD-ACE2 binding. A crystal structure of the mAb-RBD complex shows that the IF motif at the tip of 47D1 CDR H2 interacts with a hydrophobic pocket in the RBD. Diverse immunoglobulin gene usage and convergent epitope targeting characterize neutralizing antibody responses to SARS-CoV-2, suggesting that vaccines that effectively present the receptor binding site on the RBD will likely elicit neutralizing antibody responses in a large fraction of the population.

## Highlights

• 54 antibodies have neutralizing IC_50_ values in the range of 3.3–81 ng/mL
• Neutralization by 47D1 is achieved without direct blocking of RBD-ACE2 binding
• 47D1 binds only to one side of the receptor binding surface on the RBD
• Convergent epitope targeting in neutralizing antibody responses to SARS-CoV-2

## Graphical abstract

**Figure:**
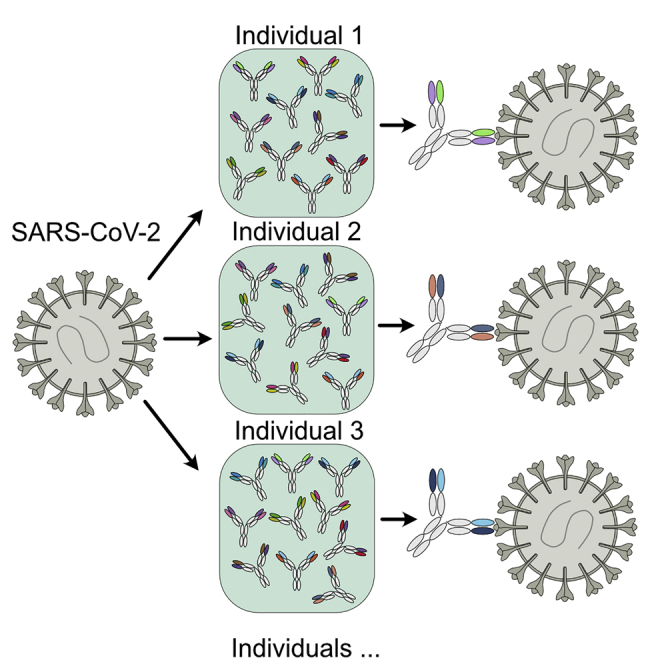

## Teaser

Zhou et al. isolate 54 potent neutralizing antibodies from convalescent COVID-19 patients, revealing diverse immunoglobulin gene usage and convergent epitope targeting of neutralizing antibody responses to SARS-CoV-2. Vaccines that effectively present the RBD receptor binding site will likely elicit neutralizing antibody responses in a large fraction of the population.

## Introduction

It is thought that humans can mount antibody responses to any non-self antigen presented to the immune system in an appropriate manner. This response is achieved by a vast repertoire of B cell receptors (BCRs). Each B cell expresses only one kind of BCR, which consists of two identical heavy chains and two identical light chains. A clonotype is a unique pair of heavy and light chains, incorporating variable (V), diversity (D), and joining (J) gene segments, but it can have distinct CDR3 amino acid sequences that include N additions, insertions, deletions, post-translational modifications, as well as somatic hypermutation. A recent deep-sequencing analysis of heavy chains of circulating B cell populations from 10 human subjects led to an estimation of the cohort-wide clonotype diversity on the order of 3 × 10^15^ (Briney et al., 2019). Because a healthy human adult has only about 5 × 10^9^ B cells in the peripheral blood, the circulating B cell population samples only a small fraction of this enormous diversity, and the subpopulation of universally shared clonotypes is likely to be small (Briney et al., 2019; Soto et al., 2019). The individuality of the B cell repertoire suggests that each person will mount a unique antibody response upon microbial pathogen infection, and that personalized vaccine delivery and therapeutic intervention may be highly beneficial in the prevention and treatment of infectious diseases (Briney et al., 2019). Nevertheless, convergent antibody responses to specific microbial pathogens have been observed, in which the same sets of immunoglobulin genes, which encode BCRs and their secreted forms (i.e., antibodies), are utilized to generate antibody responses against a given antigen in different individuals (Andrews and McDermott, 2018; Chen et al., 2019; Parameswaran et al., 2013; Pieper et al., 2017; Setliff et al., 2018). Characterization of the molecular interactions between those antibodies and their cognate antigens can facilitate rational design of vaccines aimed at eliciting antibody responses utilizing those same specific sets of immunoglobulin genes. Therefore, elucidating the individuality and convergence of antibody responses to microbial infections should provide practical frameworks to guide vaccine development.

The COVID-19 pandemic currently rampaging around the world has presented an urgent need for development of effective vaccines and antiviral medicines. First discovered in December 2019 (Zhou et al., 2020), SARS-CoV-2, the virus that causes COVID-19, has infected more than 112 million people worldwide and led to more than 2.4 million deaths as of February 23, 2021. Administration of convalescent plasma containing neutralizing antibodies to some critically ill COVID-19 patients was able to improve their clinical conditions (Duan et al., 2020; Shen et al., 2020), suggesting the beneficial effect of SARS-CoV-2 neutralizing antibodies. Monoclonal neutralizing antibody treatment has shown promising clinical outcome in multiple infectious diseases, including influenza, HIV, and Ebola (Salazar et al., 2017; Saphire et al., 2018). Therefore, it is imperative to isolate and characterize potent neutralizing antibodies for SARS-CoV-2 for potential clinical applications and to guide next-generation vaccine design.

SARS-CoV-2 and SARS-CoV-1 belong to the same viral subgenus of SARS-related CoV (SARSr-CoV), and share 79.6% nucleotide sequence identity (Zhou et al., 2020). Their entry into human cells is initiated by interaction between the receptor binding domain (RBD) of the virus spike protein (S protein) and the cell surface receptor angiotensin-converting enzyme 2 (ACE2) (Shang et al., 2020; Tai et al., 2020; Walls et al., 2020; Zhou et al., 2020). The S protein is then primed by the cellular protease TMPRSS2 and mediates fusion of viral and cellular membranes (Hoffmann et al., 2020). The functional importance of the S protein RBD makes it an important target for neutralizing antibodies. RBD is also an immunodominant target of SARS-CoV-2, and the serum levels of RBD-binding antibodies correlate strongly with SARS-CoV-2 neutralizing activity in COVID-19 patients (Premkumar et al., 2020).

As SARS-CoV-2 spreads around the world, its S protein has acquired multiple mutations, including the D614G variant, which has become the dominant form in many geographic regions (Zhao et al., 2020; https://bigd.big.ac.cn/ncov/?lang=en). More variants are emerging, including B.1.1.7 from the UK, B.1.351 from South Africa, P.1 from Brazil, and B.1.427/429 in California. Some of the mutations from these variants have been shown to reduce neutralization activity of monoclonal antibodies and sera from vaccinees and convalescent patients (Planas et al., 2021; Wang et al., 2021; Yuan et al., 2021a; Zhou et al., 2021).

In this study, we analyzed antibody responses in a cohort of 55 convalescent patients and found that their plasma-neutralizing activities decayed rapidly, with a half-life of around 2 weeks. We then isolated 54 monoclonal neutralizing antibodies from three patients, with half-maximal inhibitory concentration (IC_50_) values in the range of 3.3–80.5 ng/mL. While most of these antibodies directly block interaction between ACE2 and RBD of the SARS-CoV-2 S protein, 47D1 binds only to one side of the receptor binding surface on the RBD and exhibits highly potent neutralizing activity. Analysis of IGHV genes showed a strong positive correlation between their frequencies in SARS-CoV-2 neutralizing antibodies and in the baseline human BCR repertoires, suggesting that neutralizing antibodies against SARS-CoV-2 could be generated utilizing most IGHV genes present in B cell repertoires, and that most neutralizing antibodies target the ACE2 site on the RBD of the SARS-CoV-2 S protein. Our findings suggest that vaccines that can effectively present the ACE2 site to the immune system will likely generate neutralizing antibody responses against SARS-CoV-2 in a large fraction of the population.

## Results

### Rapid decay of SARS-CoV-2 neutralizing activity in convalescent patients

To screen convalescent patients for potent neutralizing antibody against SARS-CoV-2, we utilized a pseudovirus neutralization assay to evaluate the neutralizing activity of patient plasma samples (Rogers et al., 2020). Lentivirus expressing firefly luciferase was pseudotyped with SARS-CoV-2 S protein, of which 18 aa at the C terminus were removed to increase production of infectious virus. HeLa cells were transduced with lentivirus expressing human ACE2 (termed HeLa-ACE2 cells hereafter) to allow SARS-CoV-2 S protein-mediated virus entry and used as target cells for pseudovirus transduction.

**Figure 1:**
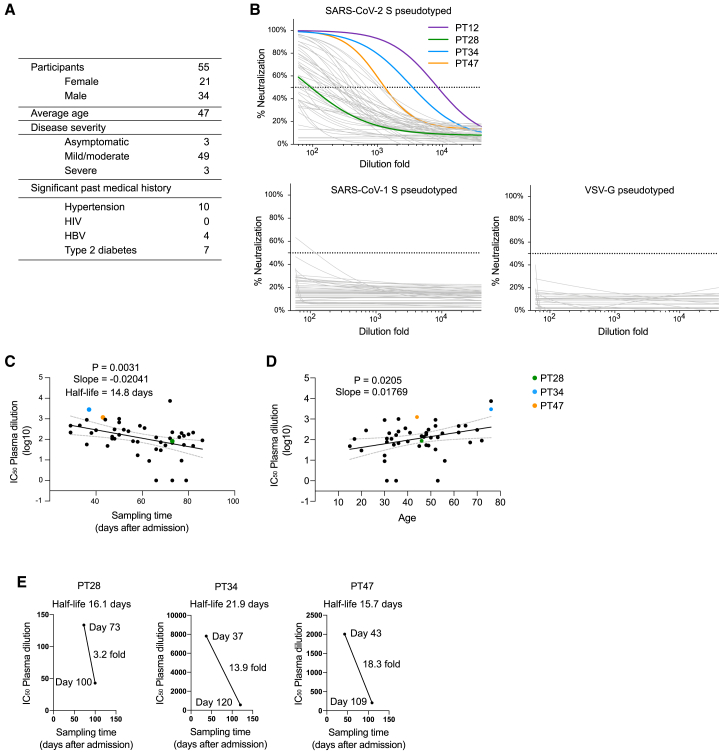
Rapid decay of plasma neutralizing activity in convalescent patients (A) Demographics of the cohort. (B) Plasma neutralizing activity of 55 convalescent patients against SARS-CoV-2 S, SARS-CoV-1 S, or VSV-G pseudotyped virus was measured using HeLa-ACE2 cells. (C) Loss of plasma neutralizing activity over time in the cohort. Sampling time indicates the time between hospital admission and sample collection. (D) Correlation of age and plasma neutralizing activity. (E) Neutralizing activity of PT28, PT34, and PT47 was measured at two different time points. Note rapid decay of plasma neutralizing activity in those three patients. Pearson correlation coefficient test was used for statistical analysis in (C) and (D). The neutralization IC_50_ values are the mean of three technical replicates.

Fifty-five donors previously infected by SARS-CoV-2 were enrolled in this study. These donors were 38% female, and the average age was 47 years (Figure 1A). All donors were immediately hospitalized and quarantined when they tested positive for SARS-CoV-2 regardless of symptoms. At the time of blood collection, all of the donors had recovered, as determined by negative SARS-CoV-2 PCR screening of nasopharyngeal swabs. Neutralization activity of the plasma was evaluated by the pseudovirus neutralization assay (Figure 1B). Lentivirus with vesicular stomatitis virus glycoprotein G (VSV-G) as the envelope protein was used as negative control to determine the suppression of SARS-CoV-2 S protein-independent virus transduction by the plasma samples. Neutralizing activity against SARS-CoV-1 was also measured using lentivirus pseudotyped with SARS-CoV-1 S protein, of which 28 aa at the C terminus were removed to increase production of infectious virus (Rogers et al., 2020). Most of the patients developed detectable neutralizing antibody against the SARS-CoV-2 S pseudotyped virus, but they exhibited very weak or no neutralizing activity against SARS-CoV-1 S or VSV-G pseudotyped virus (Figure 1B), indicating that the suppression of virus transduction by convalescent plasma is mostly specific for SARS-CoV-2 S protein. Plasma from donor PT12 showed the strongest neutralizing activity, with IC_50_ at a plasma dilution of 8,691 (Figure 1B). Notably, the neutralizing activities against SARS-CoV-2 positively correlated, albeit weakly, with patient age, but negatively correlated with the time between hospital admission and sample collection, with a half-life of 14.8 days (slope = −0.0204 log_10_) (Figures 1C and 1D), suggesting rapid decay of neutralizing antibodies in convalescent patients (Prévost et al., 2020; Seow et al., 2020). We also measured plasma neutralizing activity in donors PT28, PT34, and PT47 at a later time point. Neutralizing activity decreased by 3.2-fold from day 73 to day 100 in PT28 (half-life of 16.1 days), 13.9-fold from day 37 to day 120 in PT34 (half-life of 21.9 days), and 18.3-fold from day 43 to day 109 in PT46 (half-life of 15.7 days) (Figure 1E). The rapid loss of serum neutralizing antibodies in convalescent patients suggests that the humoral response against SARS-CoV-2 may not last very long and convalescent patients could become susceptible to re-infection of SARS-CoV-2. The neutralizing antibody response elicited by vaccination should also be evaluated for persistence.

### Isolation of neutralizing antibodies from convalescent patients

**Figure 2:**
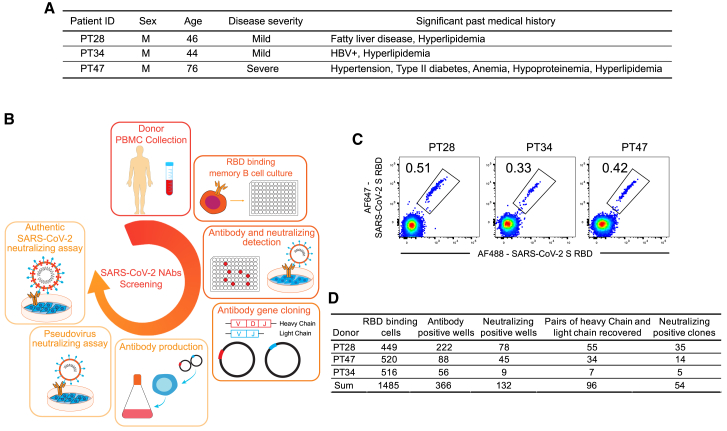
Isolation of neutralizing antibodies from convalescent patients (A) Medical history of PT28, PT34, and PT47. (B) Experiment scheme for the isolation and evaluation of neutralizing antibodies. (C) SARS-CoV-2 S RBD binding memory B cells from PT28, PT34, and PT47 were gated on CD3^−^CD4^−^CD8^−^CD14^−^CD19^+^IgD^−^IgM^−^IgG^+^ PBMCs. (D) Summary of cells and antibodies isolated from three convalescent patients. See also Table S1.

**Figure 3:**
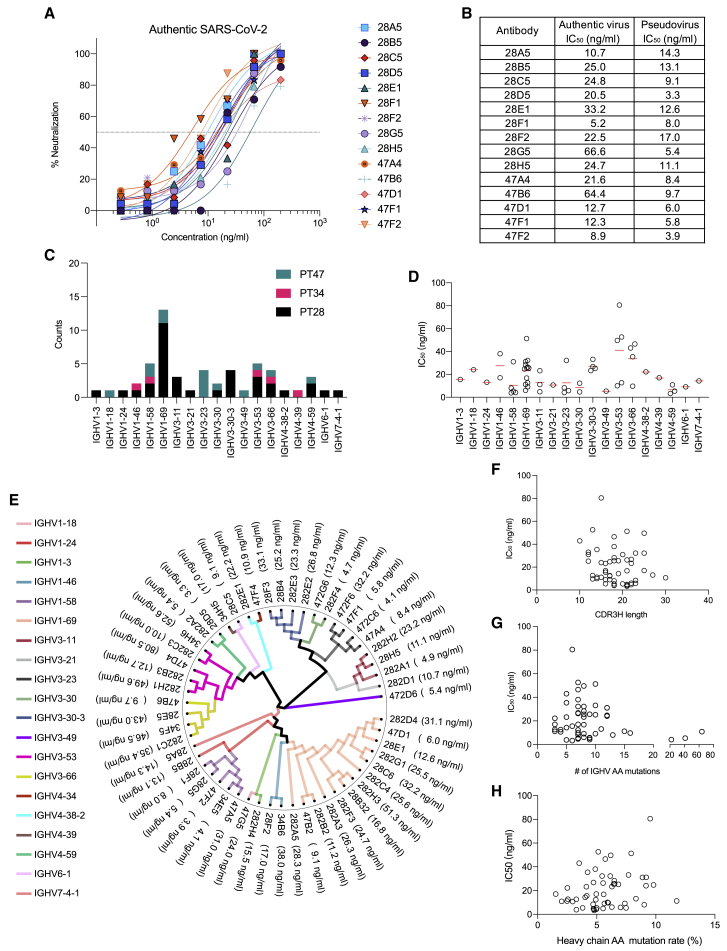
Neutralizing activity against authentic SARS-CoV-2 virus and sequence analysis of neutralizing antibodies (A and B) Neutralizing activity of selected antibodies against authentic SARS-CoV-2 virus. (C and D) IGHV gene usage of neutralizing antibodies. (E) Phylogenetic tree of neutralizing antibodies. (F–H) CDR3H length distribution (F), number of amino acid mutations (G), and amino acid mutation rate (H) of IGHV. The neutralization values are the mean of three technical replicates.

Peripheral blood mononuclear cells (PBMCs) from only three donors, PT28, PT34, and PT47, were able to be collected to isolate neutralizing antibodies (Figures 2A and 2B). While PT28 and PT34 developed only mild symptoms, PT47 developed severe COVID-19 symptoms before recovery. PBMCs were stained with Avi-tag biotinylated RBD of SARS-CoV-2 S protein to identify RBD-binding cells. 0.51%, 0.33%, and 0.42% of immunoglobulin (Ig)G^+^IgM^−^IgD^−^ B cells from PT28, PT34, and PT47, respectively, were able to bind RBD (Figure 2C). In total, 1,485 RBD-binding memory B cells were isolated and individually cultured with stromal cells in 96-well plates for 14 days, when they underwent clonal expansion and plasma cell differentiation. Culture supernatants were screened by ELISA and pseudovirus neutralizing assay to determine IgG production and neutralizing activity. Among the 1,485 wells screened, 366 wells were IgG^+^, and 132 wells of IgG^+^ showed some degree of neutralizing activity. From the 132 wells of neutralizing activity-positive cultures, 96 pairs of heavy and light chains were recovered, and their corresponding recombinant antibodies were produced using Expi293 cells (Figure 2D). 54 of 96 monoclonal antibodies showed neutralizing activity against SARS-CoV-2 S pseudotyped virus, with IC_50_ values ranging from 3.3 to 80.5 ng/mL (Table S1). Neutralizing activity against authentic SARS-CoV-2 virus of some antibodies was further evaluated (Figures 3A and 3B). Among the tested antibodies, 28F1 showed the most potent neutralizing activity against authentic SARS-CoV-2 virus, with an IC_50_ of 5.2 ng/mL.

We then characterized the immunogenetics of SARS-CoV-2 neutralizing antibodies. All antibodies were unique, although 28B4 and 28F3 shared identical CDR3s of both heavy and light chains with a few amino acid differences in non-CDR3 regions (Table S1). IGHV1-69 was the most frequently used IGHV gene in those three donors, comprising 25.9% of all neutralizing antibodies (Table S1; Figures 3C and 3E). Antibodies using IGHV4-59, IGHV1-58, and IGHV3-23 were among the most potent ones (Figures 3D and 3E). Heavy chain CDR3 length did not correlate with antibody potency (Figure 3F). Some of the most potent antibodies harbored as few as three amino acid mutations (Figure 3G), and antibody potency did not correlate with divergence from germline (Figure 3H), suggesting that extensive affinity maturation is not required to achieve a potent neutralizing antibody response to SARS-CoV-2 (Brouwer et al., 2020; Kreer et al., 2020; Liu et al., 2020; Robbiani et al., 2020; Rogers et al., 2020; Seydoux et al., 2020).

### Effective neutralization of SARS-CoV-2 variants

We next tested the neutralizing ability of these antibodies against SARS-CoV-2 variants, including the most prevalent virus, the D614G variant. The titers of viruses pseudotyped with various S protein mutants were normalized by genomic RNA quantified by qPCR. While the D614G mutation made the pseudovirus significantly more infectious, the D364Y and W436R mutations severely impaired pseudovirus infection (Figure S1A). The neutralizing ability of these antibodies against S-N354D, S-V367F, S-D614G, S-G476S, and S-V483A pseudotyped virus was measured. The G476S mutation rendered the pseudovirus more sensitive to neutralization by these antibodies, but other mutations did not significantly affect antibody neutralization in general, with a few exceptions (Figures S1B and S1C). G476S reduced the neutralizing activity of 47F2 by 11.5-fold, and V483A reduced the neutralizing activity of 47D1 by 10.9-fold (Figure S1C). D614G did not cause significant reduction in the neutralizing activity of most neutralizing antibodies (Figure S1C). Taken together, all of these antibodies effectively neutralized various SARS-CoV-2 mutants, suggesting that these mutations tested did not drive the escape of virus variants from neutralizing antibodies generated by previously infected individuals.

### 47D1 blocks spike-mediated membrane fusion

**Figure 4:**
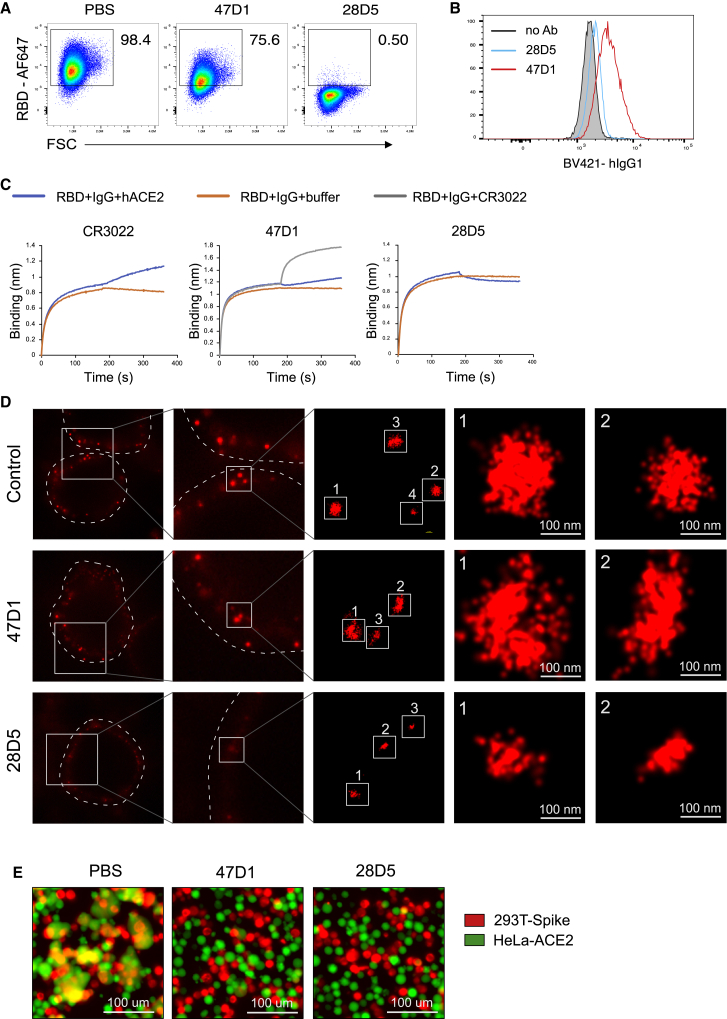
47D1 blocks spike-mediated membrane fusion (A) 47D1 or 28D5 were mixed with RBD-AF647 at a molar ratio of 2:1 and incubated with HeLa-ACE2 cells. SARS-CoV-2 RBD binding of HeLa-ACE2 cells was quantified by flow cytometry. (B) HeLa-ACE2 cells were incubated with biotinylated RBD, washed, and fixed with PFA to crosslink RBD and ACE2. Those cells were then incubated with 47D1 or 28D5, followed by incubation with BV421-anti-human IgG1 to detect antibody that bound to ACE2-interacting RBD on HeLa cells. (C) SARS-CoV-2 RBD was captured on anti-HIS biosensors and incubated with indicated antibodies, followed by incubation with ACE2 or CR3022 to determine the competition for binding sites on RBD between indicated antibodies, ACE2, and CR3022. (D) STORM images of virions on HeLa-ACE2 cell surface as detected by a SARS-CoV-2 spike S2 antibody. (E) FarRed-labeled 293T-spike cells were incubated with indicated neutralizing antibodies for 30 min, mixed 1:1 with CFSE (5-(and 6)-carboxyfluorescein diacetate, succinimidyl ester)-labeled HeLa-ACE2 for 30 min at 37°C, and imaged with a fluorescence microscope. Data are representative of two to three independent experiments. See also Figure S2.

To gain insights into the epitopes of these neutralizing antibodies, we tested whether they directly block the interaction between RBD and ACE2. Fluorophore-labeled RBD was incubated with individual antibodies at a molar ratio of 1:2. The mixture was subsequently incubated with HeLa-ACE2 cells. RBD binding of HeLa-ACE2 cells was quantified by flow cytometry to evaluate the degree of antibody block of RBD binding to ACE2. Most of these neutralizing antibodies were able to effectively block RBD binding to ACE2 on HeLa cells, with less than 2% of HeLa-ACE2 cells staining positive for Alexa Fluor 647 (AF647)-RBD (Figures 4A and S2), suggesting that they all target the ACE2 binding surface on the RBD. However, 47B2 had little effect on the RBD-ACE2 interaction (Figure S2), indicating that the epitope of 47B2 does not significantly overlap with the ACE2 binding site. 47D1 slightly suppressed RBD binding to ACE2, with most of the HeLa-ACE2 cells still staining positive for AF647-RBD (Figures 4A and S2), suggesting that the 47D1 epitope is distinct from the other antibodies here that target the ACE2 site.

We next sought to understand whether 47D1 and ACE2 could bind SARS-CoV-2 RBD simultaneously. HeLa-ACE2 cells were incubated with biotinylated RBD, washed, and fixed with paraformaldehyde (PFA) to crosslink RBD and ACE2 that were in direct contact with each other. Those cells were then incubated with 47D1 or 28D5, followed by incubation with Brilliant Violet 421 (BV421)-anti-human IgG1 to detect antibody that bound to ACE2-interacting RBD on HeLa cells. While little 28D5 bound to ACE2-interacting RBD, a significant amount of 47D1 bound to ACE2-interacting RBD on HeLa cells (Figure 4B). These results suggest that 47D1 and ACE2 can bind SARS-CoV-2 RBD simultaneously.

We next performed a biolayer interferometry competition assay to study whether the epitope of 47D1 overlaps with the CR3022 epitope. CR3022 is a neutralizing antibody previously isolated from a convalescent SARS-CoV-1 patient (ter Meulen et al., 2006). It can bind the RBDs of both SARS-CoV-1 and SARS-CoV-2 (Tian et al., 2020; Yuan et al., 2020b), and it represents the first cross-reactive epitope described for these two coronaviruses (Huo et al., 2020; Yuan et al., 2020b). In this competition assay, the SARS-CoV-2 RBD was immobilized on the biosensor tip, incubated with individual neutralizing antibodies (IgG), followed by incubation with human ACE2 or CR3022. Changes in the number of molecules bound to the biosensor tip were measured in real time. Consistent with the flow cytometry-based competition assay, 47D1 did not compete with CR3022 and only slightly competed with ACE2 (Figure 4C), suggesting that it targets a site distinct from the CR3022 epitope and the most commonly targeted component of the ACE2 binding site. As shown in Figure S1C, the V483A mutation reduced the neutralizing activity of 47D1, but not 47B2 or any of the other 52 neutralizing antibodies, by 11.5-fold. It is therefore likely that 47D1 targets an epitope harboring the V483 residue.

We utilized stochastic optical reconstruction microscopy (STORM), a *super-resolution single-molecule* imaging method, to study whether 47D1 affects virus binding to ACE2-expressing cells. Pseudovirus was incubated with neutralizing antibodies, added to HeLa-ACE2 cells, and left on ice for 1 h to allow virus attachment to the cell surface but slow down subsequent events such as membrane fusion. The HeLa-ACE2 cells were then fixed with 4% PFA, stained with an antibody recognizing SARS-CoV-2 spike S2 domain, followed by staining with an AF647-conjugated secondary antibody. The blinking signal of AF647 was captured by STORM. In the control samples, we found clustered signals on the cell membrane and the cluster sizes were about 100 nm, which is the size of pseudovirus (Figure 4D), indicating virus attachment to the cell surface. As expected, 28D5 treatment reduced the clustered signals on cell membrane. While there were still some residual signals on 28D5-treated samples, the size of those clusters was substantially smaller than the cluster size in the control samples (Figure 4D), suggesting that those are non-specific signals from the spike S2 antibody. Notably, we found clustered signals in 47D1-treated samples similar to those observed in the control samples (Figure 4D), indicating that 47D1 treatment did not affect virus attachment to ACE2-expressing cells.

We next asked whether 47D1 suppresses membrane fusion mediated by SARS-CoV-2 S protein. The fusion between HEK293T cells expressing the SARS-CoV-2 S protein (hereafter termed 293T-spike cells) and HeLa-ACE2 was used to mimic membrane fusion mediated by SARS-CoV-2 S protein. When 293T-spike and HeLa-ACE2 cells were mixed at a 1:1 ratio and cocultured for 30 min, cell fusion was evident. Importantly, pre-incubation of 293T-spike cells with either 47D1 or 28D5 effectively blocked cell fusion with HeLa-ACE2 cells (Figure 4E). Taken together, our results demonstrated that 47D1 neutralizes SARS-CoV-2 by suppressing spike-mediated membrane fusion, rather than blocking virus binding to ACE2-expressing cells.

### 47D1 protects Syrian hamsters from SARS-CoV-2 challenge

**Figure 5:**
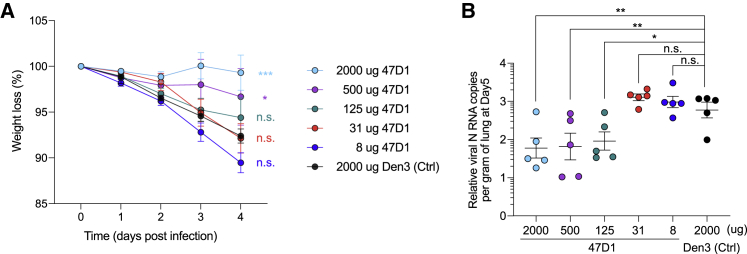
47D1 protects Syrian hamsters from SARS-CoV-2 challenge Syrian hamsters were intraperitoneally injected with 2,000, 500, 125, 31, or 8 μg/animal of 47D1, or 2,000 μg of Den3, followed by challenge intranasally 12 h post-infusion with 1 × 10^6^ PFU of SARS-CoV-2. These animals were then challenged with 1 × 10^6^ PFU of SARS-CoV-2 (USA-WA1/2020) intranasally 12 h after antibody injection. Weight loss over the course was recorded (A) and lung viral loads at day 5 were quantified by qPCR (B). Experiments were performed with five hamsters in each group. Data are represented as mean ± SEM. ^∗^p < 0.05, ^∗∗^p < 0.01, ^∗∗∗^p < 0.001, determined using a Student’s t test comparing 47D1-treated groups with the Den3 control group. n.s., not significant (p > 0.05).

We further investigated whether 47D1 protects animals from SARS-CoV-2 challenge. An unrelated antibody, Den3, was used as control. Syrian hamsters received five different doses of 47D1 at 2,000, 500, 125, 31, and 8 μg/animal intraperitoneally. These animals were then challenged with 1 × 10^6^ plaque-forming units (PFU) of SARS-CoV-2 (USA-WA1/2020) intranasally 12 h after antibody injection. Weight loss was monitored as an indication of disease severity. While the Den3-treated group showed significant weight loss after virus challenge, animals that received 2,000 or 500 μg of 47D1 showed no reduction of weight (Figure 5A). Lungs were harvested at day 5 post-infection, and viral loads in the lung tissue were measured by viral RNA qPCR. Consistent with the weight loss data, we observed significantly reduced viral titers in the lungs of animal receiving 2,000 or 500 μg of 47D1 (Figure 5B). Animals receiving 125 μg of 47D1 also showed reduced viral titer compared to the Den3 control group and displayed slightly less weight loss (Figures 5A and 5B). Therefore, 47D1 was able to protect hamsters from SARS-CoV-2 challenge.

### Diverse immunoglobulin gene usage and convergent epitope targeting of SARS-CoV-2 neutralizing antibodies

**Figure 6:**
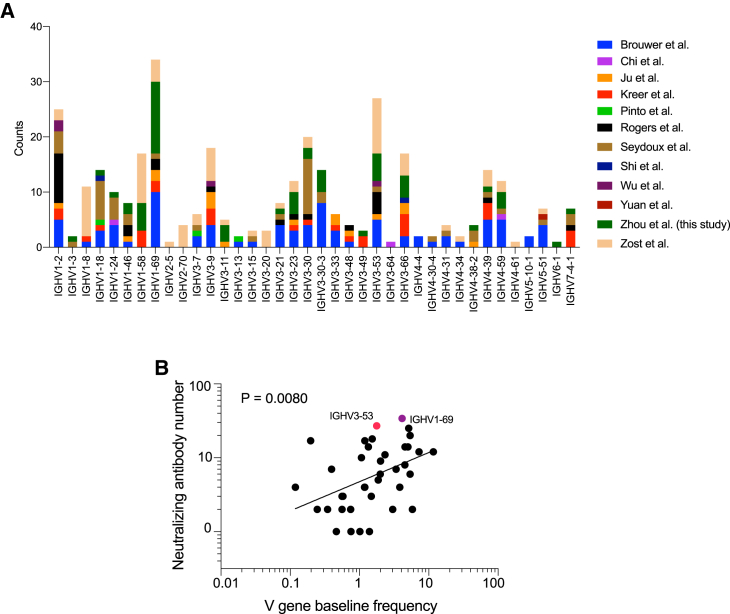
IGHV gene usage of SARS-CoV-2 neutralizing antibodies (A) IGHV genes of SARS-CoV-2 neutralizing antibodies in this and other studies (Brouwer et al., 2020; Chi et al., 2020; Ju et al., 2020; Kreer et al., 2020; Pinto et al., 2020; Rogers et al., 2020; Seydoux et al., 2020; Shi et al., 2020; Wu et al., 2020b; Yuan et al., 2020b; Zost et al., 2020b). (B) Correlation between the number of neutralizing antibodies utilizing an IGHV gene and its frequency in the baseline human BCR repertoire (Briney et al., 2019). A Pearson correlation coefficient test was used for statistical analysis. See also Tables S2 and S3.

It is quite striking that 52 of the 54 potent neutralizing antibodies isolated from three different donors directly compete with ACE2 on RBD binding. To find out whether this highly convergent neutralizing antibody response also occurred in other people infected by this coronavirus, we surveyed published studies reporting identification of neutralizing antibodies from convalescent COVID-19 donors and donors infected with SARS-CoV in 2003. Across 16 studies, including this one, almost all neutralizing antibodies were RBD binders, with most of the potent ones targeting the ACE2 binding surface on RBD (Table S2; Brouwer et al., 2020; Cao et al., 2020; Chi et al., 2020; Hansen et al., 2020; Ju et al., 2020; Kreer et al., 2020; Liu et al., 2020; Pinto et al., 2020; Robbiani et al., 2020; Rogers et al., 2020; Seydoux et al., 2020; Shi et al., 2020; Wan et al., 2020; Wu et al., 2020b; Zost et al., 2020b, 2020a). A large diversity of IGHV genes were utilized to generate those neutralizing antibodies, with a few of them (i.e., IGHV1-2, 1-69, 3-30, and 3-53/3-66) being used more frequently than others (Figure 6A). Notably, the frequencies of IGHV genes utilized in SARS-CoV-2 neutralizing antibodies showed some degree of correlation with their frequencies in the baseline human BCR repertoire (Figure 6B; Table S3). Taken together, these findings suggest that many IGHV genes can be recruited to generate neutralizing antibodies against SARS-CoV-2, with some germline genes utilized more frequently than others (Barnes et al., 2020a, 2020b; Yuan et al., 2020a), and that the most potent neutralizing antibodies often target the ACE2 binding site on the S protein of this virus.

### Structure of 47D1 in complex with SARS-CoV-2 RBD

**Figure 7:**
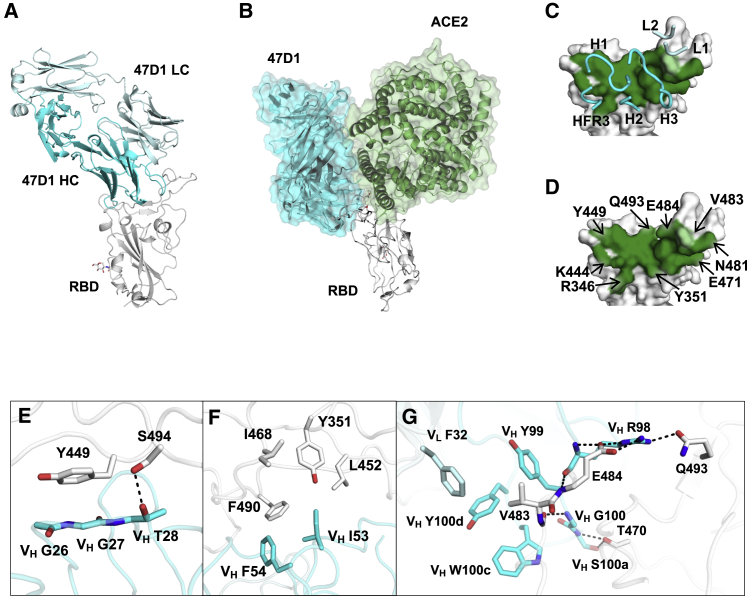
Crystal structure and key interactions of 47D1 in complex with SARS-CoV-2 RBD (A) 47D1 (cyan and pale cyan for heavy and light chains, respectively) in complex with the RBD (white). (B) 47D1 binding to RBD relative to RBD-ACE2 binding. (C and D) Epitope of 47D1. Epitope residues contacting the heavy chain are shown in dark green and those contacting the light chain are shown in light green. CDR loops and the framework region that contact the RBD are labeled. (D) Epitope residues that are important for binding to 47D1 are labeled. Epitope residues are defined here as residues in SARS-CoV-2 RBD with buried surface area (BSA) > 0 Å^2^ after Fab 47D1 binding, as calculated with Proteins, Interfaces, Structures and Assemblies (PISA) (Krissinel and Henrick, 2007). For clarity, only representative epitope residues are labeled. (E–G) Interactions between SARS-CoV-2 RBD and (E) CDR H1, (F) CDR H2, and (G) CDR H3 and L1. The heavy and light chains of 47D1 are shown in cyan and pale cyan, respectively. The RBD is in white. Hydrogen bonds are represented by dashed lines. See also Figures S3–S6 and Tables S4 and S5.

47D1 is encoded by IGHV1-69, which is the most frequently used germline gene among neutralizing antibodies identified in this study (Table S1). While numerous structures have been determined for SARS-CoV-2 antibodies, only one SARS-CoV-2 RBD-targeting IGHV1-69 antibody (LY-CoV555, also known as bamlanivimab) structure has been reported (Jones et al., 2020). To understand the molecular features of RBD recognition by IGHV1-69 antibodies, we determined the crystal structure of 47D1 Fab in complex with SARS-CoV-2 RBD to 2.1 Å resolution (Figure 7A; Tables S4 and S5). Binding of 47D1 to the RBD is dominated by its heavy chain (Figures 7A and 7C), with the heavy and light chains conferring buried surface areas (BSAs) of 716 and 34 Å^2^ on the RBD, respectively. 47D1 uses CDRs H1, H2, framework region 3 (HFR3), and H3, with minimal contributions from L1 and L2 for RBD interaction (Figures 7A and 7C). Although 47D1 appears to neutralize SARS-CoV-2 without blocking virus binding to ACE2-expressing cells (Figure 4D), 47D1 and ACE2 do overlap somewhat when we model their binding to SARS-CoV-2 RBD (Figure 7B), although the clashes with ACE2 are much less compared to other antibodies that bind to the RBS.

The high-resolution structure facilitates a detailed molecular understanding of the antibody recognition of SARS-CoV-2 RBD. The planar backbone conformation of V_H_ G26 and V_H_ G27 of CDR H1 stacks with RBD Y449, while the side chain of V_H_ T28 hydrogen bonds with RBD S494 (Figure 7E). The conserved “IF” motif in certain alleles of VH1-69 antibodies, consisting of V_H_ I53 and V_H_ F54 at the tip of CDR H2, is involved in a hydrophobic aromatic and aliphatic patch with RBD Y351, L452, I468, and F490 (Figure 7F). The CDR H3 side chains and backbone make a set of hydrogen bonds and salt bridges with RBD E484, Q493, and T470 (Figure 7G; Table S4). Together with V_L_ F32 and V_H_ Y99, W100c and Y100d of the CDR H3 also form a hydrophobic pocket with the side chain of RBD V483 (Figure 7G). V483A is a natural mutation of SARS-CoV-2 (Li et al., 2020). This mutation reduces 47D1 binding (Figure S4) and neutralization (Figure S1C), as mutation to alanine likely weakens the hydrophobic interaction.

The heavy chain of 47D1 is encoded by germline genes IGHV1-69, IGHD6-19, and IGHJ4, and the light chain is encoded by IGLV2-14 and IGLJ2. IgBlast analysis (Ye et al., 2013) indicates that IGHV of 47D1 is 3.7% somatically mutated at the nucleotide sequence level, resulting in 8 aa changes from the germline sequence, whereas IGLV is 5.1% somatically mutated, resulting in 11 aa changes (Figures S3A and S3B). Germline residue V_H_ S31 is somatically mutated to T31, where the additional γ-methyl interacts with V_H_ I53 and RBD L452, F490, and L492 (Figure S3C). The V_H_ S74F somatic mutation facilitates a T-shaped π-π stacking with RBD R346 (Figure S3D). Germline V_L_ Y32 is mutated to F32 to avoid clash with RBD V483, so that it can form a hydrophobic pocket together with CDR H3 to interact with V483 (Figure S3E). In addition, somatic mutation V_L_ E50D forms a salt bridge with V_H_ R97 in the heavy-light chain interface (Figure S3F). In short, the somatic mutations in the paratope of 47D1 suggest that they are likely involved in the affinity maturation process.

LY-CoV555 (Jones et al., 2020) also targets the receptor binding site of the SARS-CoV-2 S protein, where its binding orientation to the RBD is rotated 42° compared to that of 47D1 (calculated from the dihedral angle of the HC and LC planes), and its epitope has more overlap with the RBS compared to 47D1 (Figure S5). Previously, we classified SARS-CoV-2 RBD into three main epitopes based on antibody-complexed structures, namely RBS (sub-divided into epitopes RBS-A, RBS-B, and RBS-C), CR3022 cryptic site, and a S309 proteoglycan site (Yuan et al., 2021b). RBS-C is an epitope on the backside of the RBS on the opposite side of the RBS ridge. To date, structures of five antibodies isolated from COVID-19 patients have been reported to bind to the RBS-C epitope, namely BD-368-2 (IGHV3-23) (Du et al., 2020), P2B-2F6 (IGHV4-38-2) (Ju et al., 2020), CV07-270 (IGHV3-11) (Kreye et al., 2020), C104 (IGHV4-34) (Barnes et al., 2020a), and P17 (IGHV3-30) (Yao et al., 2021; Figure S6). In this study, we show that 47D1, an IGHV1-69 antibody, also targets RBS-C. Although these six RBS-C antibodies are encoded by different IGHV genes, their epitopes and approach angles are very similar, suggesting that the epitope is both immunogenic and the antibody binding angle and engagement enable antibody neutralization.

## Discussion

Using a high-throughput system combining isolation of RBD-binding memory B cells, *in vitro* single-cell culture, and functional screening, we isolated 54 potent neutralizing antibodies for SARS-CoV-2. Fifty-two of them target the ACE2 binding surface on the RBD of SARS-CoV-2 S protein, while the other two recognize epitopes that do not appear to overlap as extensively with the ACE2 binding site. Among the 50 or so IGHV genes, 38 were utilized to generate those neutralizing antibodies. Thus, many IGHV genes present in human B cell repertoires can be harnessed to produce neutralizing antibodies against SARS-CoV-2, with some utilized more frequently than others (Barnes et al., 2020a, 2020b; Yuan et al., 2020a). Another major feature of the antibody response to SARS-CoV-2 is that a large majority of neutralizing antibodies identified so far are RBD binders, and that the most potent ones identified to date almost always target the ACE2 binding surface on RBD of the SARS-CoV-2 S protein. In some sense, these findings are good news for vaccine development, as differences in BCR repertoires between individuals would not seem to be a major obstacle in eliciting effective neutralizing antibody responses, and vaccines effectively presenting the ACE2 binding surface of RBD to the immune system will likely generate neutralizing antibody responses against SARS-CoV-2 in a large fraction of the population.

We have determined a crystal structure of one of our neutralizing antibodies, 47D1, which utilizes the IGHV1-69 germline gene and binds a site on the backside of the receptor binding surface of the SARS-CoV-2 RBD. IGHV1-69, IGHV1-2, and IGHV3-53/3-66 are among the most frequently used germline genes encoding SARS-CoV-2 neutralizing antibodies (Figure 6A; Yuan et al., 2020a). While numerous structures have been determined for IGHV3-53 and IGHV1-2 neutralizing antibodies, only one SARS-CoV-2 RBD-targeting IGHV1-69 antibody structure has been reported to date (Jones et al., 2020). IGHV1-69-encoded antibodies have been reported to be versatile in antiviral responses to various viruses, including influenza virus, HIV-1, and hepatitis C virus (HCV) (Chen et al., 2019). Particular alleles of the IGHV1-69 gene encode two hydrophobic residues, I53 and F54, at the tip of the CDR H2 loop, which provides a structural basis for recognition of several viral epitopes. The IF motif and somatically mutated motifs facilitate binding of multiple neutralizing antibodies to a conserved hydrophobic motif in the hemagglutinin stem of influenza viruses, the MPER, HR1, and CD4bs regions of HIV-1, as well as the E2 neutralizing face of HCV (Chen et al., 2019). In the present study, we show the structural basis of an IGHV1-69 antibody targeting SARS-CoV-2, where the IF motif at the tip of CDR H2 interacts with a hydrophobic pocket in SARS-CoV-2 RBD (Figure 7F). These findings reveal a striking convergence in our immune responses to challenges by several different viral pathogens.

We investigated the mechanism of action for 47D1, which does not impair virus binding to cell surface ACE2 in our functional assays, although its epitope partially overlaps with the ACE2 binding site. Instead, it appears to suppress membrane fusion mediated by the SARS-CoV-2 S protein (Figure 4). In addition to neutralization, antibodies are able to activate many functions, such as activation of the complement system, antibody-dependent cellular cytotoxicity, antibody-dependent cellular phagocytosis, and antibody-dependent enhancement of disease (ADE) (Lu et al., 2018). While some of the potent neutralizing antibodies could mediate ADE, they are still able to protect animals from virus challenge (Kam et al., 2007; Li et al., 2021). Studies have shown that both neutralization and other effector functions of antibody contribute to optimal protection against SARS-CoV-2 challenge *in vivo* (Butler et al., 2021; Chan et al., 2020; Schäfer et al., 2021). We show in this study that 47D1 is able to protect hamsters from SARS-CoV-2 challenge. This protection could come from other effector functions of 47D1, in addition to its role in blocking SARS-CoV-2 S protein-mediated membrane fusion.

SARS-CoV-2 mutations have been increasing and new lineages are spreading worldwide. As the vast majority of existing SARS-CoV-2 neutralizing antibodies appear to target the ACE2 site, it is highly likely that virus variants capable of escape from those neutralizing antibodies will emerge. Indeed, recent studies reported that the UK variant B.1.1.7 and the South Africa variant B.1.351 are modestly or markedly more resistant to neutralization by convalescent plasma and vaccinee sera (Planas et al., 2021; Wang et al., 2021; Zhou et al., 2021). Therefore, neutralizing antibodies targeting different epitopes and vaccines designed to elicit those antibodies will play critical roles in protecting us from the next wave and lineages of SARS-CoV-2.

It is intriguing that individuals of different ages, ethnic groups, and geographical locations are all able to mount potent neutralizing antibody responses targeting a few common epitopes, such as the ACE2 binding site, considering the extraordinary clonotype diversity in the baseline human BCR repertoires and the tiny subpopulation of universally shared clonotypes (Briney et al., 2019; Soto et al., 2019). It is also striking that many IGHV genes can be utilized to generate SARS-CoV-2 neutralizing antibodies. Looking at this matter from a different angle may make it more comprehensible. The chemical essence of antibody-antigen interactions, as well as those between BCRs and antigens, are simply to engage in interactions between opposing molecular surfaces. The basic building blocks of antibodies, and most proteinaceous antigens as well, are their globular domains. Many constraints exist in the process of folding a string of amino acids into a globular domain with a well-defined structure. First, all protein structures are built up from the same three basic folding units (helices, sheets, and turns) and all structures fall into four well-defined classes (all-α, all-β, α+β, α/β) (Chothia, 1984; Levitt and Chothia, 1976). The small number of common packing classes and the limitations on chain topologies mean that certain arrangements of secondary and tertiary structures occur more frequently and that proteins with substantially different amino acid sequences may end up with highly similar backbone structures. For the sake of discussion, we would like to introduce the concept of structure clonotype, which represents a group of BCRs with similar three-dimensional structures in their antigen binding sites (i.e., paratopes). BCRs within the same structure clonotype may utilize different immunoglobulin genes and also have low CDR3 sequence similarity. Nevertheless, their antigen binding sites may adopt a similar geometry with residues capable of recapitulating key binding interactions at equivalent topological locations. Accordingly, we refer the previously defined clonotype as a sequence clonotype, which represents B cells with a unique pair of heavy and light chain amino acid sequences, including sequence variants generated by somatic hypermutation. It is conceivable that when the universe of sequence clonotypes is projected onto the universe of structure clonotypes, the diversity may drop by several orders of magnitude. Second, the BCR repertoire is sculpted by the selection processes involved in B cell development, such as heavy chain pairing with VpreB and λ5, heavy chain pairing with light chain, receptor editing, clonal deletion, and peripheral tolerance mechanisms, to secure the generation of precursor (pre-)BCRs and BCRs with signaling capacity and to purge BCRs that cross-react with self-antigens (Goodnow et al., 2005; Nemazee, 2006; Rajewsky, 1996). These constraints may further reduce the diversity of structure clonotypes by a few orders of magnitude. We speculate that the structure clonotype diversity of mature B cells in the human population is much lower than the number of mature B cells in a human adult and, therefore, there is significant sharing of structure clonotypes between individuals and in the population. In the context of antibody responses to SARS-CoV-2, there are likely multiple shared structure clonotypes targeting the ACE2 binding surface on the RBD of SARS-CoV-2 S protein, with each of them approaching the ACE2 site through different angles. This is exemplified by the most prevalent class of ACE2 site-targeting antibodies, which all utilize the IGHV3-53 gene, have short CDRH3 loops, and pair with distinct subsets of light chains. A common feature of these antibodies is that their CDRH1 and CDRH2, both encoded by the germline sequence of IGHV3-53, interact extensively with the RBD mainly through specific hydrogen bond interactions (Yuan et al., 2020a). These shared structure clonotypes may utilize many different IGHV genes, exist in a large fraction of the population, and underlie the convergent neutralizing antibody responses to SARS-CoV-2.

### Limitations of study

We recognize that it is important to define whether 47D1-like antibodies targeting the RBS-C epitope can effectively neutralize SARS-CoV-2 variants, such as B.1.1.7 from the UK, B.1.351 from South Africa, P.1 from Brazil, and B.1.427/429 in California. However, other new variants are also emerging. In this study, we were not able to test all of the variants in time. Future study is needed to identify broad neutralizing antibodies against those variants.

## STAR★Methods

### Key resources table

**Table:** 

REAGENT or RESOURCE	SOURCE	IDENTIFIER
**Antibodies**		

APC-Cy7 Anti-Human CD3 (Clone SP34-2)	BD Biosciences	Cat#557757; RRID: AB_396863
APC/Cy7 Anti-human CD4 antibody (Clone OKT4)	BioLegend	Cat#317418; RRID: AB_571947
APC-Cy7 Anti-Human CD8 (Clone RPA-T8)	BD Biosciences	Cat#557760; RRID: AB_396865)
APC/Cy7 Anti-human CD14 (Clone M5E2)	BD Biosciences	Cat#561384; RRID: AB_10611720
PerCP/Cy5.5 Anti-human CD19 (Clone HIB19)	BD Biosciences	Cat#561295; RRID: AB_10644017
PerCP/Cy5.5 Anti-human CD20 (Clone 2H7)	BioLegend	Cat#302326; RRID: AB_893283
PE anti-human IgM (Clone MHM-88)	BioLegend	Cat#305212; RRID: AB_314508
PE anti-human IgD (Clone IA6-2)	BioLegend	Cat#348204; RRID: AB_10553900
BV421 anti-human IgG (Clone G18-145)	BD Biosciences	Cat#562581; RRID: AB_2737665
F(ab’)2 goat anti-human IgG+IgM (H+L)	Jackson ImmunoResearch	Cat#109-006-127; RRID: AB_2337552
SARS-CoV-2 Spike Glycoprotein (944-1218aa) antibody	Proteintech	Cat#28867-1-AP; RRID: AB_2881223
Unconjugated goat anti-human IgG (H+L) antibody	Jackson ImmunoResearch	Cat#109-006-088; RRID: AB_2337549
Alkaline Phosphatase AffiniPure F(ab’)_2_ Fragment Goat Anti-Human IgG	Jackson ImmunoResearch	Cat#109-056-098; RRID: AB_2337618

**Bacterial and virus strains**		

SARS-CoV-2 strain USA-WA1/2020	BEI Resources	Cat#NR-52281
SARS-CoV-2 pseudotyped virus	This paper	N/A
Turbo Competent *E. coli*	NEB	Cat#C2984H

**Biological samples**		

PBMCs from COVID-19 convalescent patients	Affiliated Hospital of Putian University	N/A

**Chemicals, peptides, and recombinant proteins**		

SuperScript IV Reverse Transcriptase	Thermo Fisher	Cat#18090050
Streptavidin-AF647	Thermo Fisher	Cat# S12374
Streptavidin-AF488	Thermo Fisher	Cat# S11223
TurboCapture 96 mRNA Kit	QIAGEN	Cat# 72251
HotStarTaq DNA Polymerase	QIAGEN	Cat# 203203

**Critical commercial assays**		

Bright-Glo Luciferase Assay System	Promega	Cat# E2620

**Deposited data**		

X-ray coordinates and structure factors for 47D1/RBD	This study	PDB: 7MF1

**Experimental models: cell lines**		

HeLa cells	ATCC	Cat# CCL-2
293T cells	ATCC	Cat# ACS-450
HeLa-ACE2 cells	This study	N/A
Expi293F cells	ThermoFisher	Cat# A14527
Vero cells	ATCC	Cat# CCL-81

**Experimental models: organisms/strains**		

Golden Syrian Hamster	Charles river	N/A

**Recombinant DNA**		

pcDNA-SARS-CoV-2-S-Δ18aa	(Rogers et al., 2020)	N/A

**Software and algorithms**		

FlowJo	BD Biosciences	N/A
GraphPad Prism 8	GraphPad	N/A
PyMOL	Schrödinger, LLC	RRID: SCR_000305
Octet analysis software 9.0	Fortebio	https://www.moleculardevices.com
IMGT	International ImMunoGeneTics Information System	http://www.imgt.org
Phaser	(McCoy et al., 2007)	N/A

**Other**		

Ni-NTA biosensors	ForteBio	Cat# 18-5102
BD FACSAria Fusion Cell Sorter	BD Bioscience	N/A
STROM Super-Resolution Microscope System	Nikon	N/A

### Resource availability

#### Lead contact

Further information and requests for resources and reagents should be directed to and will be fulfilled by the lead contact, Changchun Xiao (cxiao@xmu.edu.cn).

#### Materials availability

All unique/stable reagents generated in this study are available from the Lead Contact with a completed Materials Transfer Agreement.

#### Data and code availability

X-ray coordinates and structure factors have been deposited in the RCSB Protein Data Bank with accession code PDB: 7MF1 for 47D1/RBD.

### Experimental model and subject details

#### Convalescent patients

Samples were obtained with written informed consent under a study protocol approved by the Ethics Committee of Xiamen University School of Medicine. All 55 participants (21 females and 34 males, aged between 15-76 years; Figures 1A and 2A) were recruited at Affiliated Hospital of Putian University. Participants were enrolled and allocated to single blood draws or a followed-up blood draws based on the plasma neutralization activity from the first blood draws and participants’ availability.

#### SARS-CoV-2 pseudovirus

SARS-CoV-2 spike protein pseudotyped virus was generated by transfecting HIV-based lentivirus backbone plasmid pCMV-dR8.2 dvpr (Addgene 8455), pBOB-Luciferase, and pcDNA-SARS-CoV-2-S-Δ18aa (with 18 amino acids at the C-terminal of SARS-CoV-2 spike protein removed) into 293T cells. Supernatant containing the virus was collected 48 hours after transfection and filtered through a 0.45uM filter.

#### Authentic SARS-CoV-2 virus

The authentic SARS-CoV-2 virus used was USA-WA1/2020. All experiments associated with the authentic virus were conducted in Biosafety Level 3 laboratory with standard operating procedures.

#### Animal study

The animal experiment was approved and performed in accordance with Scripps Research IACUC Protocol #20-0003. 8-week-old female Syrian hamsters were used in this study. 5 Syrian hamsters were allocated to experimental groups.

#### Cell lines

HeLa cells (human female) and 293T cells (human female) cells were maintained in DMEM containing 10% FBS (ExCell Bio), 2 mM L-glutamine, 100 U/ml penicillin, and 100 mg/ml streptomycin and incubated at 37°C in the presence of 5% CO2. HeLa-ACE2 was generated by transducing HeLa cells with lentivirus encoding human ACE2. Expi293F cells were maintained in Expi293 Expression Medium (ThermoFisher) with gentle shaking at 37°C in the presence of 8% CO2.

### Method details

#### Pseudovirus neutralization assay

SARS-CoV-2 spike protein pseudotyped virus was generated by transfecting HIV-based lentivirus backbone plasmid pCMV-dR8.2 dvpr (Addgene 8455), pBOB-Luciferase, and pcDNA-SARS-CoV-2-S-Δ18aa (with 18 amino acids at the C-terminal of SARS-CoV-2 spike protein removed) into 293T cells. Supernatant containing the virus was collected 48 hours after transfection and filtered through a 0.45uM filter. Target cell HeLa-ACE2 was generated by transducing HeLa cells with lentivirus encoding human ACE2. To evaluate the neutralizing activities of donor plasma or monoclonal antibodies, serially diluted plasma or monoclonal antibodies were mixed with the pseudovirus and incubated at 37°C for 1 hour. HeLa-ACE2 cells were then added to the mixture and cultured for 48 hours. Luciferase activity was measured using the Bright-Glo Luciferase Assay System (Promega # E2620). SARS-CoV-1 Spike protein pseudotyped virus was generated in the same way as described above, with 28 amino acids at the C terminus of the SARS-CoV-1 spike protein removed. IC_50_ was calculated using One-Site Fit LogIC_50_ non-linear regression in GraphPad Prism (San Diego, CA).

#### Authentic SARS-CoV-2 virus neutralization assay

18000 Vero cells per well were cultured in 200 μL medium in flat bottom 96 well plates overnight. On the next day, antibodies were serially diluted in 100 μL in a round bottom 96 well plates. 100 μL SARS-CoV-2 (6000 PFU per ml) was added to each well in the antibody plate and incubated at 37°C for 1 hour. Medium was then removed from Vero cells, followed by addition of 100 μL antibody/SARS-CoV-2 mixture. After incubation at 37°C for 1 hour, 100 μL methylcellulose/medium (mixed in a 1:1 ratio) was added to each well. After 3 days of incubation in a 37°C incubator, supernatant was removed. Cells were fixed with 4% PFA in PBS and stained with crystal violet for 15 minutes. Plaques were counted and the percentage of neutralization was calculated using wells without antibodies as control. IC_50_ was calculated using One-Site Fit LogIC_50_ non-liner regression in GraphPad Prism (San Diego, CA).

#### Isolation of SARS-CoV-2 RBD-specific neutralizing antibodies

Biotinylated SARS-CoV-2 RBD (Avidity BirA500) was coupled to streptavidin-AF647 (Thermo Fisher S12374) or streptavidin-AF488 (Thermo Fisher S11223) at a molar ratio of 4:1 for 30 minutes. Human peripheral blood mononuclear cells were stained with APC-Cy7 CD3 (SP34-2), APC-Cy7 CD4 (OKT4), APC-Cy7 CD8 (RPA-T8), APC-Cy7 CD14 (M5E2), PerCP-Cy5.5 CD19 (HIB19), PE IgD (IA6-2), PE IgM (MHM-88), BV421 IgG (G18-145), AF488-RBD, and AF647-RBD for 30 minutes at room temperature. RBD-AF647^+^ and RBD-AF488^+^ memory B cells (CD3^-^CD4^-^CD8^-^CD14^-^CD19^+^IgD^-^IgM^-^IgG^+^) were isolated and seeded onto 3T3msCD40L stroma cells. Cells were cultured in IMDM with 10% FBS, 100U/ml hIL-2, 50ng/ml hIL-21 for 14 days. Culture supernatants were screened for IgG production by ELISA, using unconjugated goat anti-human IgG (H+L) antibody (Jackson ImmunoResearch, 109-006-088) as capture antibody and alkaline phosphate conjugated anti-human IgG Fcγ antibody (Jackson ImmunoResearch, 109-056-098) as detection antibody. Neutralizing activity of each well was evaluated by pseudovirus neutralization assay. IgG positive and neutralizing activity positive wells were selected for antibody gene cloning.

RNA from the cultured B cells was isolated using the TurboCapture 96 mRNA Kit (QIAGEN), following manufacturer’s instructions. cDNA was generated using Superscript IV Reverse Transcriptase (Thermo Fisher) with random hexamers (Gene Link) and Ig gene-specific primers. Nested PCR amplification of heavy- and light-chain variable regions was performed using Hot Start DNA Polymerases (QIAGEN, Thermo Fisher) and previously described primer sets (Rogers et al., 2020). Second round PCR primers were modified to include additional nucleotides overlapping with the expression vectors. Paired wells were cloned in-frame into expression vectors encoding the human IgG1, Ig kappa or Ig lambda constant region. Cloned heavy- and light-chain variable regions were sequenced and subsequently analyzed using the IMGT (International ImMunoGeneTics Information System, www.imgt.org) V-quest webserver.

#### Antibody expression and purification

Antibody heavy and light chain expression vectors were transiently expressed in the Expi293 Expression System (Thermo Fisher). After 5 days of culture, 24-deep well culture supernatants were harvested and tested in the neutralization assay. Selected antibodies with potent neutralizing activity were expressed in a larger scale, and IgG was purified with Protein A Sepharose beads (GE Healthcare).

#### Stochastic optical reconstruction microscopy (STORM)

HeLa-ACE2 cells were seeded overnight on Lab-Tek II chambered cover glass. Pseudovirus was incubated with neutralizing antibodies at room temperature for 1 hour, then added to HeLa-ACE2 cells, and left on ice for 1 hour. The HeLa-ACE2 cells were then fixed with 4% PFA, stained with an antibody recognizing SARS-CoV-2 spike S2 domain (Proteintech 28867-1-AP), followed by staining with an Alexa fluor 647-conjugated secondary antibody (ThermoFisher A-21244). The blinking signal of Alexa fluor 647 was captured by STORM (Nikon).

#### Biolayer interferometry binding assay

For the binding study, the RBD construct were cloned into phCMV3 and transiently transfected into Expi293F cells using ExpiFectamine 293 Reagent (Thermo Fisher Scientific) according to the manufacturer’s instructions. The supernatant was collected at 7 days post-transfection. The RBD proteins were purified by Ni-NTA, followed by size exclusion chromatography, and buffer exchanged into 20 mM Tris-HCl pH 7.4 and 150 mM NaCl.

The N-terminal peptidase domain of human ACE2 (residues 19 to 615, GenBank: BAB40370.1) was cloned into phCMV3 vector and fused with a C-terminal Fc tag. The plasmids were transiently transfected into Expi293F cells using ExpiFectamine 293 Reagent (Thermo Fisher Scientific) according to the manufacturer’s instructions. The supernatant was collected at 7 days post-transfection. Fc-tagged ACE2 protein was then purified with a Protein A column (GE Healthcare) followed by size exclusion chromatography.

Binding kinetics assays were performed by biolayer interferometry (BLI) using an Octet Red instrument (FortéBio) as described previously (Wu et al., 2020a). Briefly, His6-tagged RBD proteins at 20 μg/ml in 1x kinetics buffer (1x PBS, pH 7.4, 0.01% BSA and 0.002% Tween 20) were loaded onto Ni-NTA biosensors and incubated with the indicated concentrations of 47D1 and CR3022 Fabs. The assay consisted of five steps: 1) baseline: 60 s with 1x kinetics buffer; 2) loading: 120 s with His_6_-tagged RBD protein; 3) baseline: 60 s with 1x kinetics buffer; 4) association: 120 s with Fabs; and 5) dissociation: 120 s with 1x kinetics buffer. For estimating the exact K_D,_ a 1:1 binding model was used.

For competition assays, CR3022 IgG, 47D1 IgG, 28D5 IgG, and human ACE2-Fc were all diluted to 200 nM. Ni-NTA biosensors were used. In brief, the assay has five steps: 1) baseline: 60 s with 1x kinetics buffer; 2) loading: 120 s with 20 μg/mL, 6x His tagged SARS-CoV-2 RBD proteins; 3) baseline: 60 s with 1x kinetics buffer; 4) first association: 180 s with CR3022 IgG, 47D1 IgG, or 28D5 IgG; and 5) second association: 180 s with human ACE2, CR3022 IgG or disassociation with 1x kinetics buffer for each first association

#### Crystal structure determination of the Fab-RBD complex

The coding sequence for receptor binding domain (RBD; residues 333-529) of the SARS-CoV-2 spike (S) protein was synthesized and cloned into a customized pFastBac vector (Ekiert et al., 2011), which is designed to fuse an N-terminal gp67 signal peptide and C-terminal His_6_-tag to the target protein. To express the RBD protein, a recombinant bacmid DNA was generated from the sequencing-confirmed pFastBac construct using the Bac-to-Bac system (Life Technologies). Baculovirus was generated by transfecting purified bacmid DNA into Sf9 cells using FuGENE HD (Promega), and subsequently used to infect suspension cultures of High Five cells (Life Technologies) at a multiplicity of infection (MOI) of 5 to 10. Infected High Five cells were incubated at 28 °C with shaking at 110 rpm for 72 hours for protein expression. RBD protein that was secreted into the supernatant was then concentrated using a 10 kDa MW cutoff Centramate cassette (Pall Corporation). The RBD protein in the concentrate was purified by affinity chromatography using Ni-NTA resin (QIAGEN), followed by size exclusion chromatography on a HiLoad Superdex 200 pg column (GE Healthcare), and buffer exchanged into 20 mM Tris-HCl pH 7.4 and 150 mM NaCl using the same protocol as before (Yuan et al., 2020b).

Purified 47D1 Fab with and SARS-CoV-2 RBD were mixed at a molar ratio of 1:1 and incubated overnight at 4°C. The complex (13 mg/ml) was screened for crystallization using the 384 conditions of the JCSG Core Suite (QIAGEN) on our custom-designed robotic CrystalMation system (Rigaku) at Scripps Research by the vapor diffusion method in sitting drops containing 0.1 μL of protein and 0.1 μL of reservoir solution. Optimized crystals were then grown in 8.5% isopropanol, 17% PEG 4000, 0.085 M HEPES pH 7.5, 15% glycerol at 20°C. Crystals were grown for 7 days, pre-equilibrated in cryoprotectant containing 10% ethylene glycol, and then flash cooled in liquid nitrogen. Diffraction data were collected at cryogenic temperature (100 K) at Brookhaven National Laboratory at beamline NSLS-II 17-ID-2 with a wavelength of 0.9793 Å, and processed with HKL2000 (Otwinowski and Minor, 1997). Structures were solved by molecular replacement using PHASER (McCoy et al., 2007) with PDB: 6W41 for RBD (Yuan et al., 2020b), whereas the model of 47D1 was generated by Repertoire Builder (https://sysimm.org/rep_builder/; Schritt et al., 2019). Iterative model building and refinement were carried out in COOT (Emsley et al., 2010) and PHENIX (Adams et al., 2010), respectively.

### Quantification and statistical analysis

Statistical analyses were performed using GraphPad Prism (San Diego, CA). IC_50_ values were determined after log10 transformation of antibody concentration using One-Site Fit LogIC_50_ non-linear regression in GraphPad Prism. Technical and biological replicates are indicated in the figure legends.

## Supplemental information

Document S1. Figures S1–S6 and Tables S1–S5

Document S2. Article plus supplemental information
