# Supplementary material for: Diverse immunoglobulin gene usage and convergent epitope targeting in neutralizing antibody responses to SARS-CoV-2
Source: Cell Rep. 2021 Apr 24;35(6):109109. doi: 10.1016/j.celrep.2021.109109 (PMC8064889; doi:10.1016/j.celrep.2021.109109)
Supplement: Document S1. Figures S1–S6 and Tables S1–S5 [file mmc1.pdf]

Cell Reports, Volume *35*

Supplemental information

Diverse immunoglobulin gene usage and convergent epitope targeting in neutralizing antibody responses to SARS-CoV-2

Xiaojuan Zhou, Fengge Ma, Jun Xie, Meng Yuan, Yunqiao Li, Namir Shaabani, Fangzhu Zhao, Deli Huang, Nicholas C. Wu, Chang-Chun D. Lee, Hejun Liu, Jiali Li, Zhonghui Chen, Yazhen Hong, Wen-Hsien Liu, Nengming Xiao, Dennis R. Burton, Haijian Tu, Hang Li, Xin Chen, John R. Teijaro, Ian A. Wilson, Changchun Xiao, and Zhe Huang

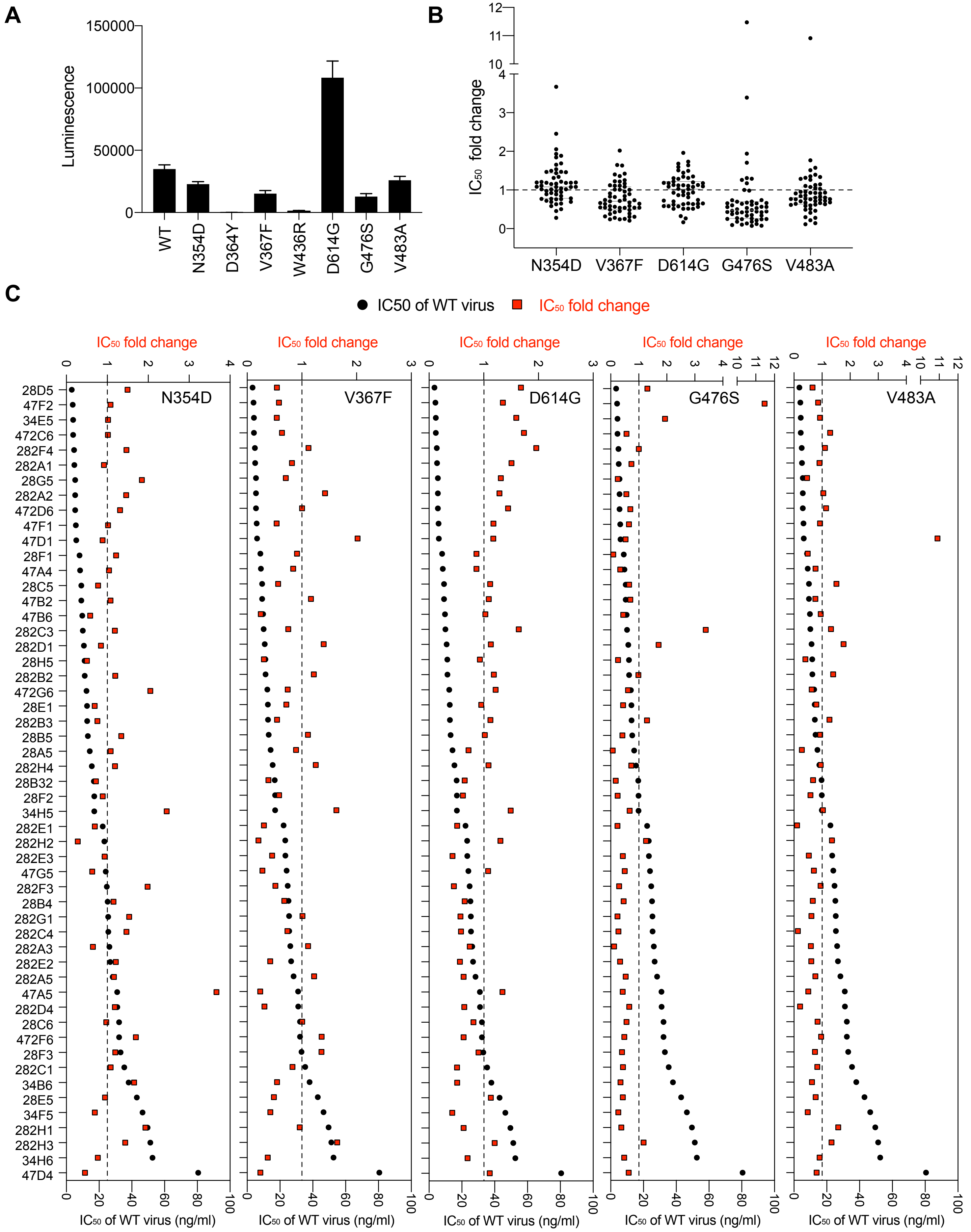

Figure S1. Neutralizing antibodies effectively neutralize various SARS-CoV-2 S variants. (A) Infection ability of lentiviruses pseudotyped with various SARS-CoV-2 S protein variants. (B, C) Fold change in neutralizing activity of antibodies against lentiviruses pseudotyped with various SARS-CoV-2 S protein variants as compared to lentiviruses pseudotyped with SARS-CoV-2 S protein from the Wuhan-Hu-1 strain (WT virus). The neutralization IC50 values are mean of three technical replicates. Related to Figure 3.

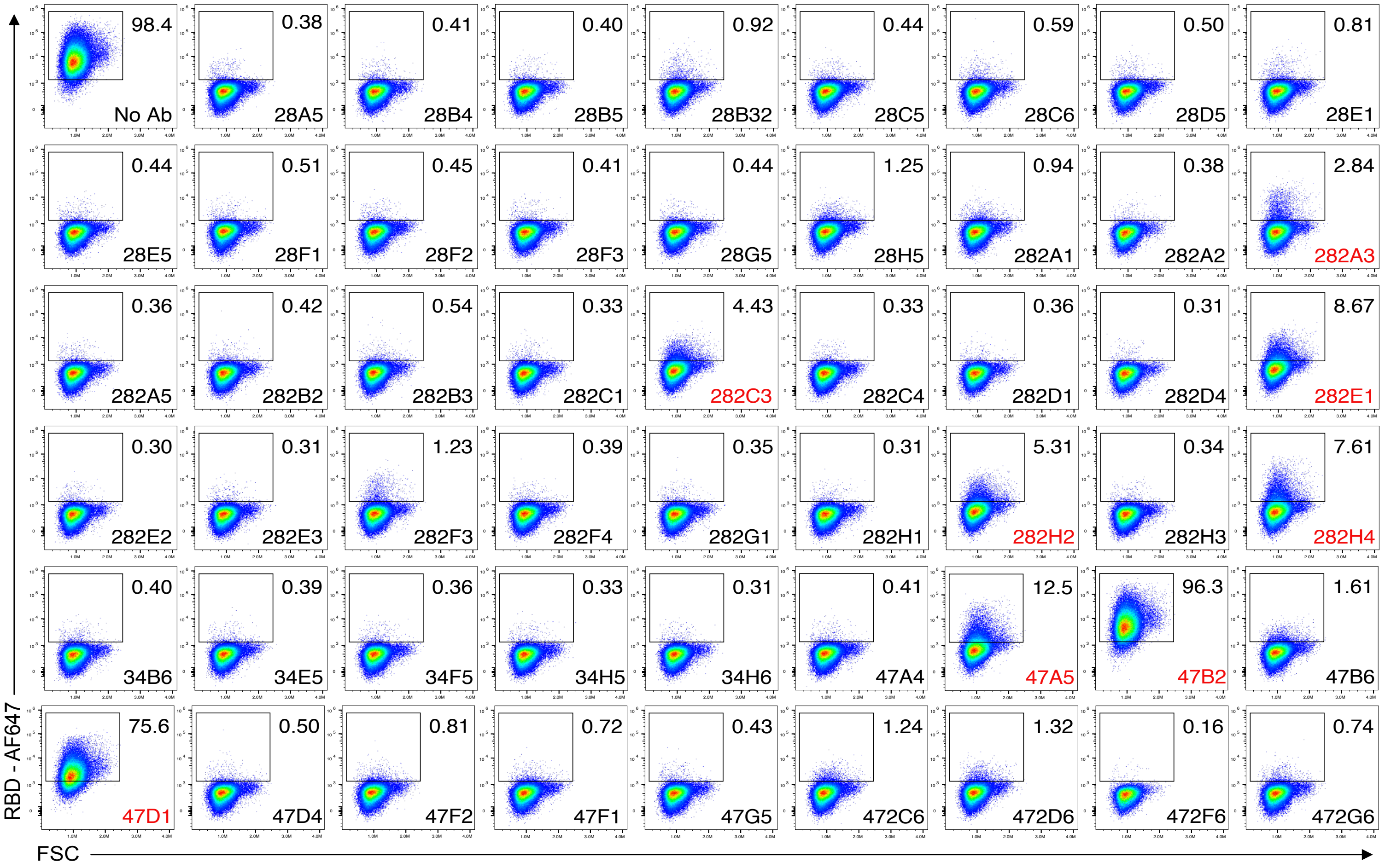

Figure S2. Flow cytometry competition assay of nAbs and RBD on ACE2. Antibodies were mixed with RBD-AF647 at a molar ratio of 2:1 and incubated with HeLa-ACE2 cells. SARS-CoV-2 RBD binding of HeLa-ACE2 cells was quantified by flow cytometry. Antibodies that did not completely block RBD binding to HeLa-ACE2 cells were marked in red. Related to Figure 4.

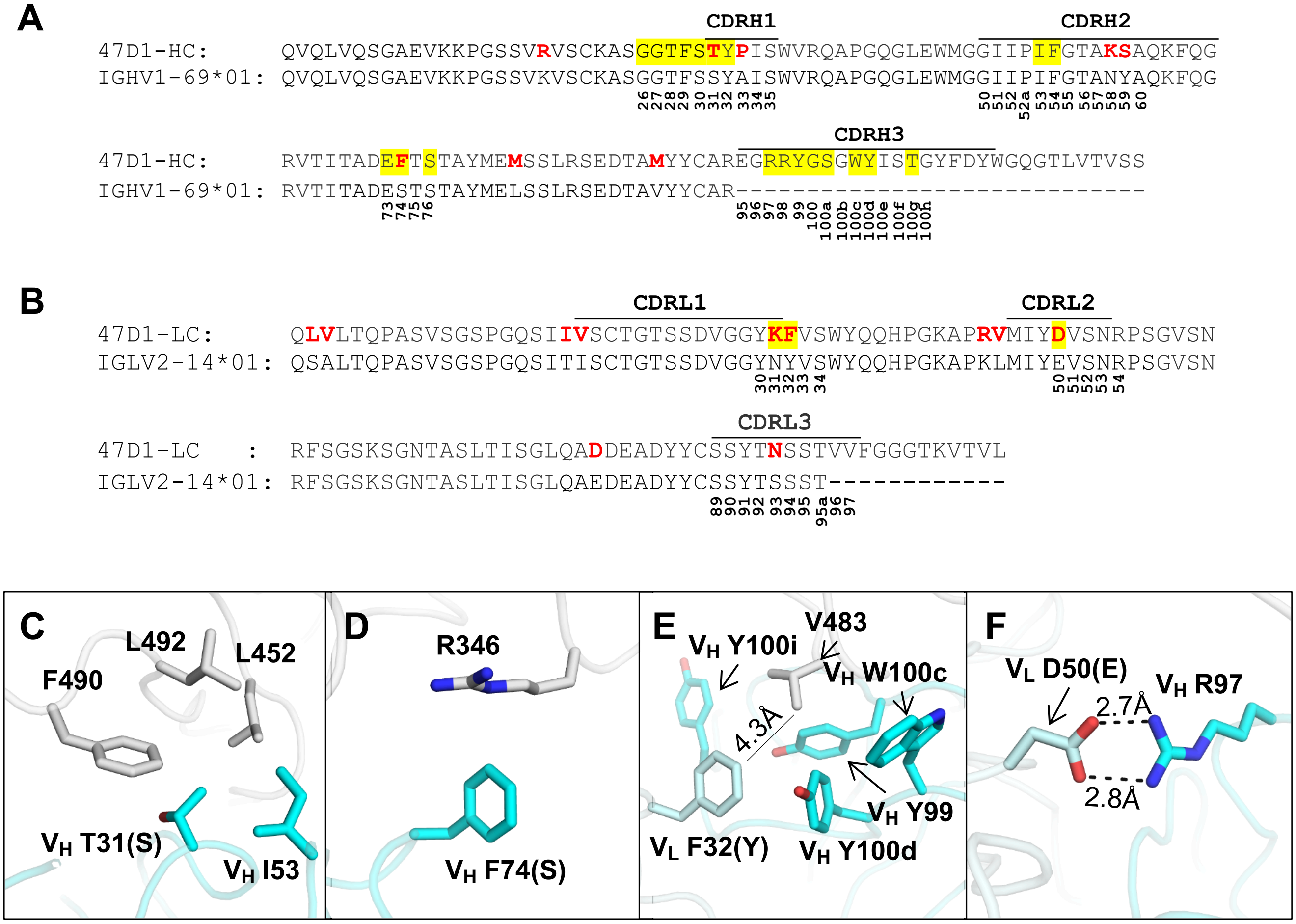

Figure S3. Putative germline sequences and somatically mutated residues of 47D1. (A-B) Alignment of 47D1 with the germline IGHV1-69*01 sequence (nucleotide SHM rate 3.7%) and IGLV2-14*01 (nucleotide SHM rate 5.1%). The regions that correspond to CDR H1, H2, H3, L1, L2, and L3 are indicated. Residues that differ from the germline are highlighted in red. Residues that interact with the RBD are highlighted in yellow. Residue positions in the CDRs are labeled according to the Kabat numbering scheme. (C-F) Somatic mutations V_H S31T, V_H S74F, V_L Y32F, and V_L E50D are located in the 47D1 paratope and other somatic mutations in the CDRs may affect overall CDR conformation and interactions. Germline residues are shown in brackets. Hydrogen bonds are represented by dashed lines. Distances between atoms are shown in solid lines. 47D1 heavy chain is in cyan and light chain is in pale cyan. SARS-CoV-2 RBD is in white. Related to Figure 7.

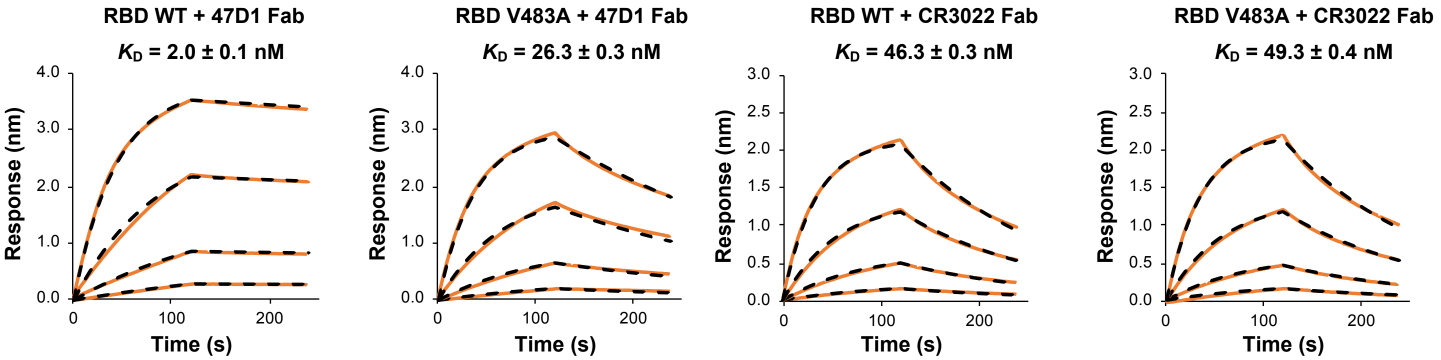

Figure S4. Sensorgrams for binding of 47D1 and CR3022 Fabs to wild-type SARS-CoV-2 RBD and a natural variant V483A. Binding kinetics were measured by biolayer interferometry with RBD proteins loaded on the biosensor and Fabs in solution. Orange solid lines represent the response curves and black dashed lines represent the 1:1 binding model. Binding kinetics were measured for four concentrations of Fab at 2-fold dilution ranging from 160 nM to 20 nM. Representative results of three replicates for each experiment are shown. Related to Figure 7.

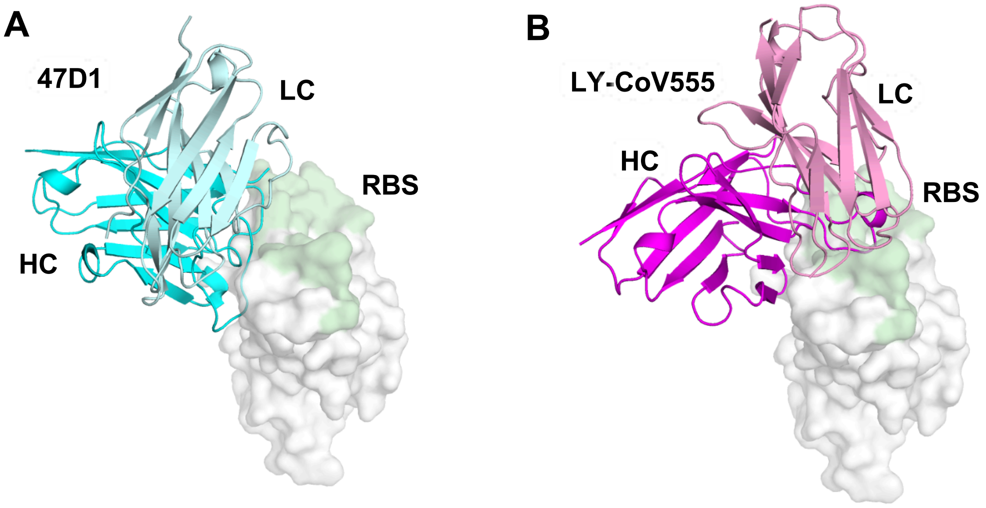

Figure S5. Comparison of IGHV1-69 antibodies 47D1 and LY-CoV555. SARS-CoV-2 RBD is shown as a white surface with the RBS in pale green. The Fv regions of the bound antibodies **(A)** 47D1 (this study) and **(B)** LY-CoV555 (PDB 7KMG, Jones et al., 2020) are represented by cyan/pale cyan and magenta/pink cartoons, respectively. Related to Figure 7.

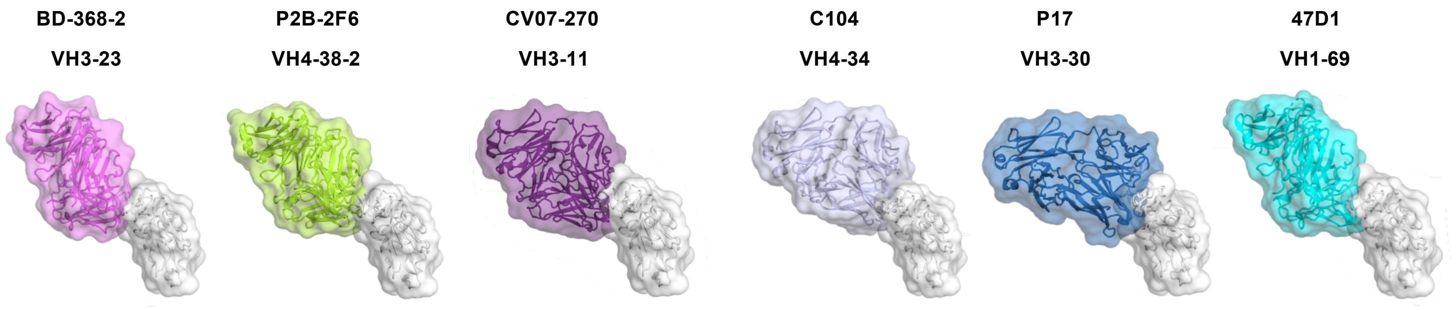

Figure S6. The epitope of 47D1 and other SARS-CoV-2 RBS-C antibodies. SARS-CoV-2 RBS-C antibodies bind to a similar epitope with a very similar angle of approach despite being encoded by distinct germline genes. Germline genes of BD-368-2 (PDB 7CHH) (Du et al., 2020), P2B-2F6 (PDB 7BWJ) (Ju et al., 2020), CV07-270 (PDB 6XKP) (Kreye et al., 2020), C104 (PDB 7K8U) (Barnes et al., 2020a), P17 (PDB 7CWO), (Yao et al., 2021) and 47D1 (this study) are analyzed by IgBLAST (Ye et al., 2013) and shown on top of each Fab-RBD complex structure, where the RBD is in white and the Fabs in different colors. Related to Figure 7.

Clone	IC_{50} (ng/ml)	Donor	Heavy Chain CDR3	Heavy Chain V	Light Chain CDR3	Light Chain V
28D5	3.3	PT28	CARYAKKTFDSESSDYHFDYW	IGHV4-59	CCSYAGSSTWVF	IGLV2-23
47F2	3.9	PT47	CAAPSCDTSICYDAFNIW	IGHV1-58	CQHYGTSIFTF	IGKV3-20
34E5	4.1	PT34	CAAPHCGGVCYDGFDVW	IGHV1-58	CQQYDRSPWTF	IGKV3-20
472C6	4.1	PT47	KRGGYCSSTICYTRYYYMDVW	IGHV3-23	CQQANSFPLTF	IGKV1-12
282F4	4.7	PT28	CARGRTYYYDSSGYYPNWFDTW	IGHV3-30	CQHYNNYPITF	IGKV1-5
282A1	4.9	PT28	CARDVDIVATIRYNYYGMDVW	IGHV3-11	CQQYDDSPPGTF	IGKV3-20
28G5	5.4	PT28	CAAPSCRGVTCYDGFNIW	IGHV1-58	CQQYDNSPWTF	IGKV3-20
472D6	5.4	PT47	CTRFQRHCSSTSCGYYMDVW	IGHV3-49	CQQYDNWLTF	IGKV3-15
282A2	5.4	PT28	CARGESWYKTSWFDPW	IGHV4-59	CTGWDDSLSGVVF	IGLV1-47
47F1	5.8	PT47	CAKRGGYCSSATCFTRFYYLDVW	IGHV3-23	CQQANSFPLTF	IGKV1-12
47D1	6.0	PT47	CAREGRRYGSGWYISTGYFDYW	IGHV1-69	CSSYTNSSTVVF	IGLV2-14
28F1	8.0	PT28	CAAPYCSGGTCYDAFDIW	IGHV1-58	CQQFGTSPWTF	IGKV3-20
47A4	8.4	PT47	CAKRGGYCTDTICYTRYYYMDVW	IGHV3-23	CQQANSFPLTF	IGKV1-12
47B2	9.1	PT47	CARREGTGWFGYGMDVW	IGHV1-69	CSSYASSSSLEVF	IGLV2-14
28C5	9.1	PT28	CARARGGTSHWDFDYW	IGHV6-1	CQQYGSSYTF	IGKV3-20
47B6	9.7	PT47	CARNLGDDAFDIW	IGHV3-66	CQQLNSYPPGTF	IGKV1-9
282C3	10.0	PT28	CARGRWEIDAFDIW	IGHV3-53	CQLLDSNPPGTF	IGKV1-9
282D1	10.7	PT28	CARELIRLGVLGDPAFYDYLYSYHYGMDVW	IGHV3-21	CCSYAGSSVVF	IGLV2-23
47F4	10.9	PT47	CAKRGGYCTDTICYTRYYYMDVW	IGHV4-59	CQSYDSSNPVVF	IGLV6-57
28H5	11.1	PT28	CATRPYYYGSGSYYW	IGHV3-11	CSSYAGNNNFELF	IGLV2-8
282B2	11.2	PT28	CARVRRPNEDALAW	IGHV1-69	CQQYHSYSPITF	IGKV1-5
472G6	12.3	PT47	CATRVMVRGVIMGFDPW	IGHV3-30	CQQSYSTPITF	IGKV1-39
28E1	12.6	PT28	CARGGDILANQDAFDFW	IGHV1-69	CQQYHSYSPLTF	IGKV1-5
282B3	12.7	PT28	CARDVPRAAGTLW	IGHV3-53	CQQYDNLPPTF	IGKV1-33
28B5	13.1	PT28	CATVDAGVIKDYFWGSYRRQGYYFDYW	IGHV1-24	CQQRSNWPWTF	IGKV3-11
28A5	14.3	PT28	CAREGYYYDSGGLVLSDYW	IGHV7-4-1	CQQYDNFRSF	IGKV1-33
282H4	15.5	PT28	CARGGQQWLVDYFEKW	IGHV1-3	CQQYHGYPWTF	IGKV1-5
28B32	16.8	PT28	CATPGGILAGPAANKDAFDIW	IGHV1-69	CQQYNSYSPITF	IGKV1-5
28F2	17.0	PT28	CAGLTAAAEPSPFDYW	IGHV1-46	CQQSYSTPPTF	IGKV1-39
34H5	17.0	PT34	CARDNADPNGSRSYYYYYYYGVDVW	IGHV4-39	CSSYTSSSTYVF	IGLV2-14
282E1	22.2	PT28	CARAVGRGVAGEF	IGHV4-38-2	CQQRSNWPPITF	IGKV3-11
282H2	23.2	PT28	CAGARGVVAGYSLSYW	IGHV3-11	CQQYDNPSLTF	IGKV1-33
282E3	23.3	PT28	CARDGLLTTMVRGIMGPAYDYW	IGHV3-30-3	CQQYDNLPYTF	IGKV1-33
47G5	24.0	PT47	CATIFSGSSYELDHW	IGHV1-18	CGTWDSSLSVMF	IGLV1-51
282F3	24.7	PT28	CATPGDVTANKDAFDVW	IGHV1-69	CQQYHSYSPITF	IGKV1-5
28B4	25.2	PT28	CARGRPRSGLYW	IGHV3-30-3	CQQYDEPTLTF	IGKV1-33
282G1	25.5	PT28	CARGDLTASGSGSDGMDVW	IGHV1-69	CQQYHSYSPITF	IGKV1-5
282C4	25.6	PT28	CARSAYYSDSSGYHFDYW	IGHV1-69	CQQYHSSPRTF	IGKV3-20
282A3	26.3	PT28	CATPGSIVGMSTTNKDAFDIW	IGHV1-69	CQQYHSYSPITF	IGKV1-5
282E2	26.8	PT28	CARDGESITMVRGLMSPAFDYW	IGHV3-30-3	CQQRYNWPRTF	IGKV3-11
282A5	28.3	PT28	CASPPGYTNSWYQFFQRW	IGHV1-69	CQQYNSYSYTF	IGKV1-5
47A5	31.0	PT47	CAASVRITDFDWFEDGFDIW	IGHV1-58	CQQFGSSPYTF	IGKV3-20
282D4	31.1	PT28	CARVSNGGNDYW	IGHV1-69	CQAWDSSTALYVF	IGLV3-1
28C6	32.2	PT28	CATPADILAGSDAGPGKGTDTFDFW	IGHV1-69	CQQYKSYSPLTF	IGKV1-5
472F6	32.2	PT47	CAKRGGYCTTTICYTRYYYMDVW	IGHV3-23	CQQANSFPLTF	IGKV1-12
28F3	33.1	PT28	CARGRPRSGLYW	IGHV3-30-3	CQQYDEPTLTF	IGKV1-33
282C1	35.4	PT28	CARFGYGTSRYDYW	IGHV3-66	CQQYDNLPVTF	IGKV1-33
34B6	38.0	PT34	CASGSLAPNFFYYAMGVW	IGHV1-46	CSSWDSSDNHPIF	IGLV3-19
28E5	43.0	PT28	CARDLRDWVW	IGHV3-66	CHQYDNLPRTF	IGKV1-33
34F5	46.5	PT34	CARDYYASGSYGWADSGW	IGHV3-66	CQQYITWPPMYTF	IGKV3-15
282H1	49.6	PT28	CARVARDLRVMYGDLNYYYYGMDVW	IGHV3-53	CQLTYTIPRF	IGKV1-39
282H3	51.3	PT28	CARATLGDCSGGPCGDAFDIW	IGHV1-69	CQQYHSYSPLTF	IGKV1-5
34H6	52.6	PT34	CARDLGPYGMDVW	IGHV3-53	CQQLNSYPPYTF	IGKV1-9
47D4	80.5	PT47	CARDPYCRGGGCHIW	IGHV3-53	CQQYDNLPITF	IGKV1-33

Table S1. Activity, immunoglobulin gene usage, and CDR3 amino-acid sequences of neutralizing antibodies. CDR3s with identical amino-acid sequences are highlighted in the same color. Related to Figure 2 and 3.

Publications	Isolated neutralizing antibodies and their epitopes
Brouwer et al., Science, 2020	19 neutralizing antibodies (nAbs) isolated. 14 of them bind RBD. At least 5 RBD binding nAbs target ACE2 site.
Cao et al., Cell, 2020	14 nAbs isolated. The most potent one binds ACE2 site
Chi et al., Science, 2020	5 antibodies neutralize pseudotyped virus and 3 of them neutralize authentic virus. One of the five nAbs targets ACE2 site and another one (4A8) targets NTD.
Hansen et al., Science, 2020	Isolated over 200 antibodies with some degree of neutralizing activity. The most potent 9 nAbs all target ACE2 site.
Ju et al., Nature, 2020	16 nAbs isolated. 13 of them bind ACE2 site. The most potent antibodies (P2C-1F11, P2B-2F6, and P2C-1A3) bind ACE2 site.
Kreer et al., Cell, 2020	28 nAbs isolated. 27 of them bind RBD.
Liu et al., Nature, 2020	19 potent nAbs isolated. nAbs directed against the top of RBD compete strongly with ACE2 binding and potently neutralize the virus. Those directed against the side surfaces of RBD do not compete with ACE2 and neutralize less potently.
Pinto et al., Nature, 2020	Isolated S309 from SARS patient, targeting non-ACE2 site
Robbiani et al., Nature, 2020	52 isolated nAbs bind RBD at three epitopes (not further characterized).
Rogers et al., Science, 2020	25 nAbs isolated. 24 of them bind two epitopes on RBD: RBD-A, RBD-B. Most potent nAbs target RBD-A (ACE2 site).
Seydoux et al., Immunity, 2020	2 nAbs isolated. The most potent one targets the ACE2 site.
Shi et al., Nature, 2020	2 nAbs isolated. CB6 binds ACE2 site.
Wan et al., Cell Reports, 2020	11 nAbs isolated. 6 of them target ACE2 site.
Wu et al., Science, 2020	4 nAbs isolated. 2 most potent ones target ACE2 site.
Zhou et al., 2020 (this study)	Isolated 54 antibodies. 52 of them target ACE2 site.
Zost et al., Nature Medicine, 2020<br>Zost et al., Nature, 2020	70 nAbs isolated. 67 of them bind RBD. Among the 40 nAbs further characterized , 38 (including the most potent ones) target ACE2 site.

Table S2. SARS-CoV-2 neutralizing antibodies and their epitopes. Related to Figure 6.

IGHV genes	Baseline frequency (%) Briney et al.	Brouwer et al.	Chi et al.	Ju et al.	Kreer et al.	Rogers et al.	Pinto et al.	Seydoux et al.	Shi et al.	Wu et al.	Yuan et al.	Zhou et al. (this study)	Zost et al.	Subtotal
IGHV1-2	5.13	5		1	2	9		4		2			2	25
IGHV1-3	0.56							1				1		2
IGHV1-8	2.34	1			1								9	11
IGHV1-18	4.54	3			1		1	7	1			1		14
IGHV1-24	1.07	4	1					4				1		10
IGHV1-45	0.01													
IGHV1-46	2.01	1		1		3		2				2		9
IGHV1-58	0.20				3							5	9	17
IGHV1-69	4.12	10		2	2	2		1				13	4	34
IGHV1-69-2	0.07													
IGHV2-5	0.46												1	1
IGHV2-26	0.01													
IGHV2-70 or IGHV2-70D	0.12												4	4
IGHV3-7	5.37	2					1	1					2	6
IGHV3-9	1.53	4		3	3	1				1			6	18
IGHV3-11	1.86			1								3	1	5
IGHV3-13	0.35	1					1							2
IGHV3-15	1.48	1						1					1	3
IGHV3-20	0.58												3	3
IGHV3-21	4.50	4				1		1				1	1	8
IGHV3-23	11.66	3		1	1	1						4	2	12
IGHV3-30	5.37	4			1	1		10				2	2	20
IGHV3-30-3	1.34	8						2				4		14
IGHV3-33	1.99	3		2	1									6
IGHV3-43 or IGHV3-43D	0.45													
IGHV3-48	3.81	1		1	1	1								4
IGHV3-49	0.56				2							1		3
IGHV3-53	1.77	5		1		4		1		1		5	10	27
IGHV3-64 or IGHV3-64D	0.75		1											1
IGHV3-66	1.20	2		2	4				1			4	4	17
IGHV3-72	0.31													
IGHV3-73	0.37													
IGHV3-74	2.97													
IGHV3-NL1	0.05													
IGHV4-4	3.01	2												2
IGHV4-28	0.03													
IGHV4-30-2	0.54													
IGHV4-30-4	0.74	1						1						2
IGHV4-31	1.20	2						1					1	4
IGHV4-34	5.80	1											1	2
IGHV4-38-2	1.18			1				2				1		4
IGHV4-39	5.01	5			3	1		1				1	3	14
IGHV4-59	7.22	5	1					1				3	2	12
IGHV4-61	1.39												1	1
IGHV5-10-1	0.25	2												2
IGHV5-51	3.37	4						1			1		1	7
IGHV6-1	1.01											1		1
IGHV7-4-1	0.40				3	1		2				1		7

Table S3. Numbers of neutralizing antibodies utilizing individual IGHV genes. Related to Figure 6.

Data collection	47D1 + SARS-CoV-2 RBD
Beamline	NSLS-II 17-ID-2
Wavelength (Å)	0.9793 Å
Space group	P 2 2 2
Unit cell parameters	
a, b, c (Å)	75.7, 81.2, 112.5
α, β, γ (°)	90, 90, 90
Resolution (Å) [a]	50.0-2.10 (2.14-2.10)
Unique reflections [a]	41,701
Redundancy [a]	5.8 (3.0)
Completeness (%) [a]	98.0 (85.4)
$<I/\sigma_I>$ [a]	38.0 (1.0)
R_{sym}[b] (%) [a]	9.5 (90.7)
R_{pim}[b] (%) [a]	2.8 (37.8)
$CC_{1/2}$[c] (%) [a]	99.5 (69.4)
**Refinement statistics**	
Resolution (Å)	2.14-2.10
Reflections (work)	41,608
Reflections (test)	2,005
R_{cryst}[d] / R_{free}[e] (%)	20.7/25.0
No. of atoms	4,979
RBD	1,536
Fab	3,271
Glycan	52
Solvent	120
Average *B*-values (Å^2)	63
RBD	68
Fab	60
Glycan	94
Solvent	58
Wilson *B*-value (Å^2)	56
**RMSD from ideal geometry**	
Bond length (Å)	0.003
Bond angle (°)	0.61
**Ramachandran statistics (%)**	
Favored	96.5
Outliers	0.0
**PDB code**	7MF1

[a] Numbers in parentheses refer to the highest resolution shell.

[b] $R_{sym} = \Sigma_{hkl} \Sigma_i | I_{hkl,i} - <I_{hkl}> | / \Sigma_{hkl} \Sigma_i I_{hkl,i}$ and $R_{pim} = \Sigma_{hkl} (1/(n-1))^{1/2} \Sigma_i | I_{hkl,i} - <I_{hkl}> | / \Sigma_{hkl} \Sigma_i I_{hkl,i}$, where $I_{hkl,i}$ is the scaled intensity of the ith measurement of reflection h, k, l, $<I_{hkl}>$ is the average intensity for that reflection, and *n* is the redundancy.

[c] $CC_{1/2}$ = Pearson correlation coefficient between two random half datasets.

[d] $R_{cryst} = \Sigma_{hkl} | F_o - F_c | / \Sigma_{hkl} | F_o |$ x 100, where F_o and F_c are the observed and calculated structure factors, respectively.

[e] R_{free} was calculated as for R_{cryst}, but on a test set comprising 5% of the data excluded from refinement.

Table S4. X-ray data collection and refinement statistics. Related to Figure 7.

47D1	Distance [Å]	SARS-CoV-2 RBD
**Hydrogen bonds**		
H:SER100A[OG]	3.7	A:THR470[O]
H:SER100A[N]	3.1	A:THR470[O]
H:GLY100[N]	3.0	A:GLY482[O]
H:ARG98[NH2]	3.3	A:GLU484[OE1]
H:ARG98[NE]	2.7	A:GLU484[OE2]
H:ARG98[N]	2.6	A:GLU484[OE2]
H:ARG98[NH1]	3.8	A:LEU492[O]
H:THR28[OG1]	2.6	A:SER494[OG]
H:PHE74[O]	3.8	A:LYS444[NZ]
H:ARG98[O]	3.1	A:GLU484[N]
**Salt bridges**		
H:ARG98[NE]	3.7	A:GLU484[OE1]
H:ARG98[NH2]	3.3	A:GLU484[OE1]
H:ARG98[NE]	2.7	A:GLU484[OE2]
H:ARG98[NH2]	3.7	A:GLU484[OE2]

Table S5. Hydrogen bonds and salt bridges identified at the 47D1-RBD interface using the PISA program. Related to Figure 7.
